# Pharmacists Knowledge, Attitudes, and Practices Regarding Probiotics and Prebiotics: A Cross-Sectional Study from Palestine

**DOI:** 10.1371/journal.pone.0350648

**Published:** 2026-06-18

**Authors:** Dala N. Daraghmeh, Leen Kahla, Noor Taqatq

**Affiliations:** Pharmaceutical Research and Innovation Hub, Department of Pharmacology, Faculty of Pharmacy, Al-Quds University, Jerusalem,‌‌ West Bank,‌‌ Palestine; University of Jeddah, SAUDI ARABIA

## Abstract

**Background:**

Probiotics and prebiotics are increasingly used globally as part of complementary health intervention for various disorders. Pharmacists, as accessible healthcare providers, are key in guiding safe and effective use. However, little is known about Palestinian pharmacists’ knowledge, attitudes, and practices (KAP) towards these products. This study aimed to assess pharmacists’ KAP regarding probiotics and prebiotics in Palestine and to identify demographic and professional factors associated with pharmacists’ knowledge score.

**Methods:**

A cross-sectional study was conducted among 400 pharmacists using a self-administered structured questionnaire, covering socio-demographics, probiotics and prebiotics knowledge, attitudes, and practices. Knowledge was assessed using a scoring system, and participants were categorized as having poor (≤50%; 0–17 points), moderate (>50%– < 70%; 18–23 points), or high (≥70%; 24–34 points) knowledge. Descriptive statistics, chi-square tests and multivariable linear regression were used for analysis. All analyses were performed using SPSS version 26.

**Result:**

Among 400 participating pharmacists, the majority of participants were female (66.25%), aged between 20 and 29 (74.25%), were staff pharmacists (82.50%), and worked in community pharmacies settings (92.25%). The mean knowledge score was 21.3 ± 6.1 (range: 4–34). Most participants demonstrated moderate knowledge (57.8%), while 32.0% achieved high knowledge and 10.3% had poor knowledge. In multivariable analysis, selected factors showed modest associations with knowledge score, including age, probiotic/prebiotic education, and prescription volume (P < 0.05). Attitudes were generally positive, with 85.5% believed they provide health benefits and 81% were willing to recommend them. Practice findings indicated variable self-reported counseling and recommendation behaviors, with barriers related to evidence access and confidence in clinical application.

**Conclusion:**

Pharmacists in Palestine showed moderate overall knowledge, with positive attitudes towards probiotics and prebiotics, yet knowledge gaps persist. These findings suggest a need for targeted practice-oriented education and continuing professional development to strengthen evidence-based counseling and appropriate clinical application.

## Introduction

The human gut microbiota plays a fundamental role in maintaining homeostasis by regulating digestion, immunity, and metabolic functions [1]. Disruption in this microbial balance, known as dysbiosis, has been associated with various conditions, including gastrointestinal disorders, metabolic diseases, and immune dysfunctions [2]. In this context, probiotics- defined by the World Health Organization as “live microorganisms which, when administered in adequate amounts, confer a health benefit on the host” [3], have gained growing clinical and scientific attention.

Certain probiotic strains (e.g., *Lactobacillus* and *Bifidobacterium*) have been investigated for potential benefits in digestive health and immune modulation, and emerging research has explored possible links with mental well-being through the microbiota–gut-brain axis [4]. Evidence syntheses suggest potential benefits for selected gastrointestinal outcomes (e.g., irritable bowel syndrome symptoms, and antibiotic-associated diarrhea) [5–7]. However, the clinical effects of probiotics are not uniform across products, and reported benefits may vary by strain(s), dose, formulation, and target condition; therefore, the evidence should be interpreted critically and applied in an indication-specific manner.

Prebiotics are non-digestible substrates that are selectively utilized by host microorganisms and may confer health benefits [8]; they are naturally present in foods such as chicory root, garlic, and onions, which promote the growth of beneficial gut bacteria [9].

The fermentation of prebiotics by gut microbes produces short-chain fatty acids, that may improve gut barrier function and exert anti-inflammatory effects [10]. The combination of probiotics and prebiotics is referred to as synbiotics, which may further enhance the survival and activity of beneficial microorganisms in the gut [11]. Synbiotics have shown potential in enhancing gastrointestinal health, modulating immune responses, and metabolic conditions [12].

The global market for probiotic products has expanded substantially, increasing the availability of these supplements in pharmacies and healthcare settings. Dietitians are the primary professionals responsible for nutritional assessment and counseling and gastroenterologists often lead care for gastrointestinal disorders [13]. However, probiotics and prebiotics are widely available as over-the-counter (OTC) foods and dietary supplements and are frequently sought by the public without prior consultation. Therefore, pharmacists, as highly accessible and commonly approached for OTC product advice, are well positioned to guide the safe and evidence-based use of probiotics and prebiotics, including appropriate product selection and safety screening [11,14].

However, studies from various countries highlight knowledge gaps among pharmacists concerning their mechanisms, suitable indications, and evidence-based applications, which may limit their ability to provide effective patient counseling and promote rational usage [15,16].

Despite growing public interest and availability of these products in the Palestinian market, little is known about pharmacists’ knowledge, attitudes, and clinical engagement with probiotics and prebiotics. Understanding these aspects is essential to inform continuing education, strengthen evidence-based counseling, and promote rational integration of microbiome-related interventions into pharmacy practice. Therefore, this study aimed to assess the knowledge, attitudes, and practices (KAP) regarding probiotics and prebiotics in Palestine, and to identify demographic and professional factors associated with pharmacists’ knowledge score.

## Methods

### Study design

A cross-sectional descriptive study was conducted in Palestine, from 20/12/2024–15/05/2025, to assess Palestinian pharmacists’ KAP regarding probiotics and prebiotics and identify demographic and professional factors influencing their knowledge. This study was designed and reported in accordance with the Strengthening the Reporting of Observational Studies in Epidemiology (STROBE) guidelines for cross-sectional studies.

### Study population, sampling procedure and sample size calculation

All licensed pharmacists working either in community or hospital pharmacies across Palestine, were eligible to take part in this study. According to the 2024 records from the Palestinian Pharmacy Association, there are 7771 registered pharmacists. The minimum target sample size was calculated to be 367, which includes a 5% margin of error and a 95% confidence interval. To determine the required sample size accurately, a software program (Raosoft sample size calculator: http://www.raosoft.com/samplesize.html) was used. Participants were recruited using a non-probability online approach (convenience sampling with snowball dissemination) through professional pharmacist networks, including WhatsApp groups and social media platforms.

The inclusion criteria encompassed practicing pharmacists in community or hospital pharmacies. Pharmacists were excluded if they were retired, not currently practicing or declined to participate.

### Survey design and validation

After a comprehensive literature review, a self-administered questionnaire was created, comprising both open and closed-ended questions. The questions used were in line with previous studies [6,7,13,16–19] and refined to fit the Palestinian practice context (including addition, removal, and rewording of items as needed). Content validity was assessed by a purposively selected four expert panel invited from academic and clinical settings based on relevant expertise in probiotics/prebiotics and pharmacist counseling. The panel comprised four members: one practicing pharmacist, one nutritionist, and two clinical pharmacologists. They rated item relevance on a 4-point scale (1 = not relevant, 2 = somewhat relevant, 3 = quite relevant, 4 = highly relevant). The scale-level Content Validity Index (CVI) was 0.82, indicating strong agreement and high content validity. Afterward, a pilot study (n = 20) was conducted by approaching pharmacists in person. Participants completed the draft questionnaire electronically using Google Forms to assess clarity, comprehension, and completion time. Feedback resulted in minor refinements, and pilot responses were excluded from the final dataset and analyses.

The finalized questionnaire was organized into five distinct sections (S1 File):

- **The first section** includes questions on sociodemographic characteristics, such as gender, age group, professional role, educational level, years of experience, working hours per week, geographic location, and province.
- **The second section** evaluates knowledge of probiotics and prebiotics, as the familiarity with probiotic/prebiotic concepts, proper terminology and knowledge of microbial species that contain probiotic strains. Responses are based on multiple choice, multiple selection and “Correct”, “Incorrect” or “I don’t know” answers.
- **The third section** evaluates practice – related questions including how often with which health care workers provide nutritional counsel and recommend probiotics in their professional activities. The responses are measured using a Likert scale ranging from “Never” to “Always”.
- **The fourth section** assesses attitudes and practices towards recommending probiotics such as reasons for advising or not advising probiotics, situations for which probiotics are recommended and barriers to prescribing them. Responses are based on multiple selection answers.
- **The fifth section** looks into the source of information about probiotics and prebiotics including prior education on probiotics, preferred future information sources (e.g., scientific journals, social media, symposia) and the desired type of information (e.g., efficacy, safety, mechanism of action).

### Survey distribution and administration

The final survey instrument was distributed through a self-administered Google Form. From December 2024 to May 2025, the survey link was shared with the targeted eligible participants via email, WhatsApp messages, and text messages.

A variety of strategies and approaches were employed to identify and recruit appropriate participants, including: Direct outreach to community pharmacists by the research team and posting the survey link on social media platforms linked to Palestinian pharmacists’ groups.

### Statistical analysis

Following data collection, data were extracted and logged in an Excel workbook (Microsoft office version, 2013). Data cleaning, coding and grouping were performed prior to analysis. Descriptive and inferential analyses were conducted to summarize pharmacists’ KAP toward probiotics and prebiotics and to examine factors associated with pharmacists’ knowledge score. Descriptive statistics were used to summarize all variables collected through the questionnaire by calculating the frequency (%) for binary variables and the median with interquartile range (IQR) for continuous variables.

Knowledge assessment of probiotics and prebiotics was evaluated through a scoring system, which categorized responses into levels to calculate a knowledge score. Correct answers were assigned one point, while incorrect responses or omissions received no points. Participants were categorized into three knowledge levels based on their total scores:

1. Poor Knowledge: 50% or less (≤ 17 points)
2. Moderate Knowledge: Scores between >50% and <70% (18–23 points)
3. High Knowledge: Scores of 70% or higher (24–34 points)

Associations between knowledge level categories and sociodemographic/professional characteristics were examined using Pearson’s Chi-Square test or Fisher’s exact test, as appropriate. Statistical significance was set at p-value < 0.05. To identify independent factors associated with pharmacists’ knowledge score, multivariable linear regression was performed with knowledge score as the dependent variable. Variables were selected a priori based on conceptual relevance to knowledge acquisition and evidence form prior KAP literature, focusing on pharmacist characteristics and practice-related factors, rather than automated stepwise selection. These included probiotic/ prebiotic education, age, gender, prescription volume per day, and practice setting. Years of experience was not included in the final model due to conceptual overlap with age and to maintain model parsimony.

All categorical variables were entered as factors and dummy-coded, allowing regression coefficients to be interpreted as comparisons between categories relative to defined reference groups. Knowledge score was treated as a continuous variable, and linear regression was considered appropriate based on the approximately continuous nature of the composite score and inspection of model assumptions. Complete-case (listwise deletion) analysis was used for the multivariable model. To assess potential bias due to complete-case analysis, included and excluded participants were compared across key variables using chi-square and t-tests. Residual diagnostics (including assessment of linearity, homoscedasticity, and normality) were performed and did not indicate major violations of model assumptions, although minor deviations at the tails were observed. As a sensitivity analysis, robust standard errors (HC1) were applied to account for potential heteroscedasticity, and results remained consistent with the primary model (S1 Table).

Attitude-related items were measured using a 5- Likert scale, positively (strongly agree, agree), neutral, negatively (strongly disagree, disagree). For reporting, Likert-scale responses were collapsed into three categories (agree/strongly agree, neutral, disagree/strongly disagree), and practice items were summarized as frequencies and percentages. All analyses were performed using SPSS version 26.

Missing data were present for a small number of variables. Descriptive statistics were calculated using all available responses, and missing values were reported where applicable. For multivariable linear regression, a complete-case (listwise deletion) approach was used; therefore, only respondents with complete data for all variables included in the model were analyzed (complete-case regression sample size was reported in the Results/Table footnote).

### Ethics approval and consent to participate

Ethical approval for the study was obtained by the Research Ethics Committee of Al-Quds University (Archived number: 457/REC/2024). Informed consent was obtained electronically prior to participation. Before accessing the questionnaire, participants reviewed the study information and indicated consent by selecting ‘Yes, I agree to participate’ (mandatory item); those who selected ‘No’ could not proceed to the survey. No direct identifiers were collected, and responses were analyzed in de-identified form. All methods were performed in accordance with relevant guidelines and regulations.

## Results

### Sociodemographic characteristics

A total of 400 pharmacists participated in this study. Due to the various recruitment methods used including social media, determining the response rate was not feasible. The sociodemographic characteristics of the participants are presented in Table 1.

**Table 1:** Sociodemographic Characteristics of participating pharmacists (n = 400).

Category	Total n = 400 n(%)
**Gender**	
Male	135 (33.75)
Female	265 (66.25)
**Age (years)**	
20-29	297 (74.25)
30-39	63 (15.75)
40-49	25 (6.25)
≥50	15 (3.75)
**Profile**	
Manager	28 (7.00)
Owner	42 (10.50)
Staff Pharmacist	330 (82.50)
**Working Settings**	
Community pharmacies	369 (92.25)
Hospital pharmacies Outpatient	11 (2.75)
Hospital pharmacies inpatient	20 (5.00)
**Educational Level**	
Bachelor of Pharmacy	327 (81.75)
Pharm D	35 (8.75)
Master	31 (7.75)
PhD	7 (1.75)
**Years of Experience**	
less than one year	100 (25.00)
1-5 years	240 (60.00)
>5–10	16 (4.00)
More than 10 years	44 (11.00)
**Working Hours/Week**	
24 hrs or less	129 (32.25)
25–40 hrs	170 (42.50)
More than 40 hrs	101(25.25)
**Residency**	
City	262 (65.50)
Village	122 (30.50)
Camp	16 (4.00)
**Pharmacy Open Hours/Week**	
Less than 80 h	74 (18.50)
80–120 h	263 (65.75)
>120h/week	63 (15.75)
**Prescriptions/Day**	
less than 50	245 (61.25)
50 and more	103 (25.75)
Missing	52(13.00)
**Employees at Site**	
< 5	299 (74.75)
≥5	48 (12.00)
Missing	53 (13.25)
**Have you ever used probiotic for yourself**	
Yes	161 (40.25)
No	239 (59.75)

The majority of participants were female (66.25%), aged between 20 and 29 (74.25%), staff pharmacists (82.5%), and worked in community pharmacy settings (92.25%).

Regarding educational background, 81.75% held a Bachelor of Pharmacy degree, while 8.8% had a PharmD, 7.8% a Master’s, and 1.8% a PhD. In terms of professional experience, 60% had worked for 1–5 years, 25% for less than one year, and 11% had more than 10 years of experience. Geographically, most pharmacies were located in cities (65.5%), followed by villages (30.5%) and Palestinian refugee camps (4.0%). Most pharmacies operated 80–120 hours per week (65.8%), with 15.8% open 24/7. Additionally, 70.4% handled fewer than 50 prescriptions per day, and 86.2% worked in practices with fewer than five employees, reflecting small-scale operations. Among the surveyed pharmacists, 40% (n = 161) reported using probiotics, while the majority (60%, n = 239) did not (Table 1).

### Education and sources of information

The findings indicate that a significant portion of pharmacists had not received specialized education on probiotics and prebiotics (Table 2). Overall, 57% reported no formal training, 26.00% indicated they had received some education, and 17% were unsure of their knowledge. Despite these gaps, the majority (93.3%) expressed interest in learning more (Table 2), specifically about clinical applications, with efficacy (90%) and safety (55.5% believing they were “not at all dangerous”) being top priorities.

**Table 2:** Current information sources and future educational Interest of pharmacists regarding Probiotics and Prebiotics.

	n (%)
**Have you ever received any specific education on Probiotic/ prebiotics?**	
Yes	104 (26.00)
No	228 (57.00)
Unsure	68 (17.00)
**Source of current information**	
Internet	212 (53.00)
Discussion with coworkers	170 (42.50)
Scholarly articles	117 (29.25)
Book	108 (27.00)
Medical education	68 (17.00)
Distributed material through my department	56 (14.00)
Lecture by members of my department	38 (9.50)
Mandatory training modules	16 (4.00)
Radio or TV	14 (3.50)
Self-learning	2 (0.50)
Others	12 (3.00)
**Interest in learning more n = 400)**	
Yes	373 (93.33)
No	27 (6.75)
**Preferred Source of Future Information (n = 333)**	
E-learning	150 (45.05)
Newsletter	70 (21.02)
Scientific journals	60 (18.01)
Lay journal (public)	30 (9.01)
Social media	28 (8.41)
Symposia	24 (7.21)
Email	18 (5.41)

Current information sources were mainly the internet (53%) and discussions with coworkers (42.5%), followed by scholarly articles (29.3%) and books (27%). Less frequently used were departmental materials (14%), medical education (17%), and lectures (9.5%) (Table 2). Preferred future sources of information included e-learning (45.05%), scientific journals (18.01%), and newsletters (21.02%), with lower preference for social media, symposia, and lay journals (Table 2).

### General Knowledge Assessment regarding probiotics and prebiotics

The responses to the Knowledge questions regarding probiotics and prebiotics are presented in S2 Table. Overall, 89.0% of participants correctly defined probiotics, 67.0% correctly defined prebiotics, 81.0% correctly defined synbiotics, and 65.0% recognize the gut microbiome. When asked about the functional benefits of probiotics, a substantial majority recognized their roles in treating gastrointestinal disorders (90.0%), improving oral health (80.0%), and supporting vaginal health (75.0%). Moreover, 76.0% believed probiotics could serve as alternatives to antibiotics, and 62.0% agreed they could enhance immune function. However, awareness of other indications was limited, with only 23.0% supporting their use in reducing UTIs and less than half recognizing possible advantages for cardiovascular health (47.0%), respiratory immunity (46.0%), or allergy relief (44.0%). Misconceptions were also common—50% incorrectly believing probiotics only work in capsules, powder or tablet form.

In terms of knowledge about prebiotics (Table 3), 72.0% of pharmacists recognized their health benefits, and 69.0% were aware of their role in selectively stimulating the growth of beneficial bacteria. However, only 42.0% correctly understood that prebiotics are resistant to degradation by gastric acid and digestive enzymes in the human gastrointestinal tract.

**Table 3:** Responses for Knowledge Assessment of Prebiotics among participants (n = 400).

Knowledge item- prebiotics	Correct	Incorrect	Not sure
Not degraded by human GIT acid or enzyme	171(42.75)	72(18.00)	157(39.25)
Fermented by micorbiota	217(54.25)	43(10.75)	140(35.00)
Confers health benefit to host	288(72.00)	33(8.25)	79(19.75)
Selectively increases good bacteria	276(69.00)	41(10.25)	83(20.75)
Shelf unstable	146(36.50)	78(19.50)	176(44.00)
Help feed the probiotics which can help build immunity	271(67.75)	33(8.25)	96(24.00)
Decrease absorption of calcium and magnesium	65(16.25)	117(29.25)	218(54.50)
Reduced triglyceride in hypercholesterolemia	132(33.00)	60(15.00)	208(52.00)

**Fig 1:**
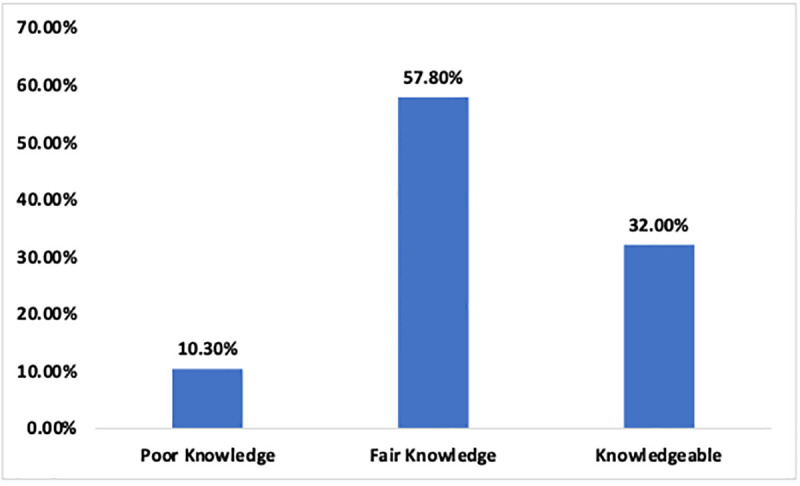
Knowledge Levels‌‌ of Participants.

The overall mean knowledge score among all participants (n = 400) was 21.27 ± 6.05, with scores ranging from 4.00 to 34.00. Most participants (231(57.8%)) demonstrated moderate knowledge, while (128(32.0%)) exhibited high knowledge, and (41(10.3%)) had a poor knowledge (Fig 1).

Pharmacists demonstrated varying awareness of microorganisms recognized as probiotics (Table 4). The majority correctly identified *Lactobacillus acidophilus* (86.8%), while less than half selected *Bifidobacterium bifidum* (46.8%) and *Lactobacillus rhamnosus* (46.5%). Fewer participants recognized *Bacillus subtilis* (33.8%), *Enterococcus faecium* (24.0%), or *Saccharomyces boulardii* (20.0%). Misidentifications were also noted, with 22.5% selecting *Escherichia coli* and 16.5% *Mycobacterium avium*, which are not generally considered probiotic species.

**Table 4:** Knowledge of microorganism’s strains.

Select species from the below list of microorganisms that you believe contain probiotic strains.	
*Lactobacillus acidophilus*	347 (86.75)
*Bifidobacterium bifidum*	187 (46.75)
*Mycobacterium avium*	66 (16.50)
*Escherichia coli*	90 (22.50)
*Lactobacillus rhamnosus*	186 (46.50)
*Bacillus subtilis*	135 (33.75)
*Enterococcus faecium*	96 (24.00)
*Saccharomyces boulardii*	80 (20.00)

### Associations of knowledge levels and sociodemographic variables and professional characteristics

S3 Table shows the relationship between the participants’ knowledge scores and their sociodemographic characteristics. Chi-square testing showed that knowledge level differed significantly by prescriptions handled per day (p = 0.044) and by receipt of specific education on probiotics/prebiotics (p < 0.001), while no significant differences were observed for gender (p = 0.197), age (p = 0.297), profile (p = 0.676), working setting (p = 0.879), educational level (p = 0.531), years of experience (p = 0.073), working hours/week (p = 0.120), residency (p = 0.172), pharmacy open hours/week (p = 0.551), number of employees (p = 0.791), or personal probiotic use (p = 0.154). Among those with >10 years of experience, 47.7% were classified as having high knowledge. Poor knowledge was more common among pharmacists handling ≥50 prescriptions/day (13.6%) compared with <50/day (5.7%). Knowledge level also varied across categories of specific education on probiotics/prebiotics (p < 0.001).

### Multivariable regression analysis

A multivariable linear regression model was fitted with knowledge score as the dependent variable. Predictor variables included probiotic/prebiotic education, age, gender, prescription volume per day, and practice setting. All categorical variables were entered as factors and dummy-coded, allowing coefficients to be interpreted as comparisons with reference categories.

The model demonstrated limited but statistically significant explanatory power (R² = 0.06; adjusted R² = 0.04, p = 0.003).

After adjustment for all covariates, probiotic education, age, and prescription volume were significantly associated with knowledge score. Pharmacists who reported prior education on probiotics/prebiotics had significantly higher knowledge scores (B = 2.00, p = 0.005). Age was also associated with knowledge score, with pharmacists aged 20–29, 30–39, and 40–49 years had significantly higher knowledge scores compared to those aged ≥50 years (all p < 0.05). In addition, pharmacists handling fewer than 50 prescriptions per day had higher knowledge scores (B = 1.68, p = 0.02). Gender, and practice setting were not significantly associated with knowledge score (p > 0.05 for both) (Table 5).

**Table 5:** Multivariable linear regression analysis of facrors associatie with knowledge score.

Variable	Category	B (SE)	p-value
**Probiotic education**	Yes vs No	2.00 (0.70)	0.005
**Age (years)**	20–29 vs ≥ 50	4.21 (1.84)	0.023
	30–39 vs ≥ 50	4.72 (1.94)	0.016
	40–49 vs ≥ 50	5.48 (2.21)	0.014
**Gender**	Male vs Female	−0.50 (0.72)	0.49
**Prescriptions per day**	<50 vs ≥ 50	1.68 (0.72)	0.020
**Practice setting**	Hospital vs Community	−0.91 (1.32)	0.49

Multicollinearity diagnostics indicated no evidence of collinearity among predictors (all VIF values ≈1).

The multivariable regression analysis included 348 participants with complete data, while 52 participants (13.0%) were excluded due to missing values. Comparisons between included and excluded participants showed no statistically significant differences in gender (p = 0.74), age group (p = 0.62), probiotic/prebiotic education (p = 0.28), or practice setting (p = 0.17). Additionally, mean knowledge scores did not differ significantly between groups (21.22 vs 21.62, p = 0.60). Because missingness in prescription volume contributed directly to case exclusion, comparison for this variable was not informative. Overall, these findings suggest limited evidence of substantial selection bias.

### Attitude assessment and practices

Table 6 summarizes pharmacists’ attitude and practice regarding probiotic and prebiotics. The majority of pharmacists believed that probiotics improve gut health (80.3%), help prevent gastrointestinal diseases (77.0%), support immunity (75.5%), and benefit overall health (85.5%). Willingness to recommend was high (81.0%), though only half (50.0%) considered them safe for all populations.

**Table 6:** Attitudes and practices of Pharmacists toward Probiotics and Prebiotics (n = 400).

Attitude Statement	Agree/Strongly Agree (%)	Neutral (%)	Disagree/Strongly Disagree (%)
Probiotics and prebiotics help improve gut health	321 (80.25%)	58 (14.50%)	21 (5.25%)
They help in preventing gastrointestinal diseases	308 (77.00%)	72 (18.00%)	20 (5.00%)
They support the immune system	302 (75.50%)	79 (19.75%)	19 (4.75%)
They are beneficial for overall health	342 (85.50%)	42 (10.50%)	16 (4.00%)
They are safe for all populations	200 (50.00%)	146 (36.50%)	54 (13.50%)
I am willing to recommend them to patients	324 (81.00%)	54 (13.50%)	22 (5.50%)

Regarding practice, 70.0% had recommended probiotics, most commonly for IBS (72.5%), improved digestion (76.1%), bloating (69.6%), diarrhea (62.5%), and post-antibiotic use (63.9%). Recommendations were often driven by patient requests, perceived efficacy, prior experience, and guideline adherence (Table 7).

**Table 7:** Pharmacists practices regarding probiotics and prebiotics.

Have you ever recommended that your patients use probiotics?	
Yes	280 (70.00%)
No	120 (30.00%)
**How often do you advise probiotics in your practice?**	
Never	44 (11.0%)
Rarely	91 (22.8%)
Sometimes	161 (40.3%)
Often	80 (20.0%)
Always	24 (6.0%)
**How often do you give nutritional advice in your practice?**	
Never	40 (10.00)
Rarely	60 (15.00)
Sometimes	133 (33.25)
Often	134 (33.50)
Always	33 (8.25)
**Drivers for Advising using probiotic/ prebiotics (n = 280)**	
Patients request	144(51.43)
insufficient efficacy of other therapies	108(38.57)
Following of colleagues’ advice	53(18.93)
Good experience with probiotics	120(42.86)
Adherence to guideline	96(34.29)
No harm no foul	78(27.86)
Evidence on probiotic efficacy	160(57.14)
As part of healthy lifestyle	129(46.07)
**Indication for probiotic advice n = 280**	
For a patient During and/or after a course of antibiotics	179(63.93)
For IBS symptoms	203(72.50)
For diarrhoea	175(62.50)
If a patient is run down and/or susceptible to common infections	64(22.86)
Constipation	125(44.64)
Diverticulitis	30(10.71)
Ulcerative colitis	113(40.36)
Before travel abroad	53(18.93)
Older people	71(25.36)
For mothers or babies in families with tendency to allergy	38(13.57)
For improved digestion	213(76.07)
To reduce bloating	195(69.64)
Dermatological problems	40(14.29)
Dental health	42(15.00)
Stress	25 (8.93)
**Reason for not recommending probiotics/ prebiotics n = 120**	
Little or no idea about probiotics.	70(58.33)
There are no clinical applications for probiotics in my specialty	32(26.67)
Not convinced of clinical benefit	20(16.67)
Cost	27(22.50)
Negative experiences with prior use	17(14.17)
Lack of knowledge about Clinical use of Probiotics	12(10.00)
Lack of information regarding available probiotics products	48(40.00)
Limited or non-availability of clinically proven probiotics products	22(18.33)
Clinical use of probiotics is controversial.	14(11.67)
Doubt in the quality of probiotics products	8(6.67)
No data on safety of probiotics.	16(13.33)
Dietary supplements, like probiotics, are not regulated by the FDA	15(12.50)
Traditional yogurts are as effective as probiotics products.	19(15.83)
The efficacy of probiotics is inferior to, or does not provide additional benefit over standard therapeutics	6(5.00)
Risk of infection due to probiotics‘use	18(15.00)

Among non-advising pharmacists (30.0%), barriers included limited knowledge (58.3%), lack of product information (40.0%), cost (22.5%), and safety or regulatory concerns.

## Discussion

This study explored pharmacists’ KAP and factors associated with their knowledge of probiotics and prebiotics in Palestine. The results showed that the majority of pharmacists possessed moderate knowledge with substantial variability in knowledge levels, highlighting potential gaps in professional awareness and competency that may influence their ability to provide evidence-based recommendations. The multivariable analysis identified modest associations between selected factors and knowledge score. Age was significantly associated with knowledge score, with younger pharmacists demonstrating higher knowledge levels, which may reflect more recent exposure to updated educational content and emerging topics such as microbiome-related therapies. Given the descriptive nature of KAP studies, the regression analysis was intended to explore potential associations rather than to develop a predictive model. Although the regression model demonstrated modest explanatory power, this is consistent with KAP studies, where knowledge is influenced by multiple unmeasured behavioral, educational, and contextual factors not fully captured in survey-based designs. Additional factors such as continuing professional education and access to evidence resources likely contribute to knowledge differences.

Attitudes toward probiotics were generally positive with most participants believing that probiotics are beneficial to human health, and expressing willingness to recommend them when supported by appropriate scientific evidence. However, a notable portion of pharmacists still held misconceptions or safety concerns regarding the use of probiotics in clinical practice. This matters in Palestine because pharmacists are often the first point of contact for OTC supplement advice; strengthening pharmacists’ evidence-based knowledge supports patient safety, rational supplement use, and more appropriate counseling alongside antimicrobial stewardship goals (e.g., avoiding inappropriate recommendations in vulnerable groups and encouraging evidence-aligned use when indicated).

The assessment of attitudes provided important insights into pharmacists’ perceptions and their willingness to integrate probiotics into patient care. Identifying these perceptions is crucial because attitudes often shape clinical decision-making and influence whether pharmacists actively recommend probiotics to patients. Understanding these attitudes can help inform targeted educational programs that address misconceptions and increase confidence in recommending probiotics in clinical settings. In addition to knowledge and attitudes, the practice component captured pharmacists’ self-reported counseling and recommendation behaviors and perceived barriers, underscoring the need for practice-oriented training focused on evidence appraisal and clinically appropriate use.

Our findings are consistent with similar studies worldwide, but also highlight regional differences. For example, studies in other countries such as the United States or some European nations have reported higher levels of knowledge among pharmacists [12,15], possibly due to differences in pharmacy education curricula, stronger emphasis on microbiome science in professional training, and the presence of structured continuing education programs in high income countries. In contrast, studies from the Middle East report similar findings [20,21]. For example, a study in the United Arab Emirates reported that pharmacists knew the general benefits of probiotics but lacked knowledge on strain-specific effects and indications [21]. Similarly, research in Saudi Arabia and Jordan reported limited understanding of safety and dosing despite positive attitudes [20,22].

The strong positive attitude toward probiotics found in this study aligns with global trends [12,15], reinforcing that pharmacists recognize the therapeutic potential of probiotics. However, the misconceptions observed, particularly around safety and efficacy, highlight an educational gap not consistently reported in similar studies. This suggests that while attitudes are globally optimistic, the depth of knowledge and application in practice may vary based on regional healthcare policies, availability of products, and professional development opportunities.

Another key finding is reliance on informal, easily accessible information sources such as the internet or peer discussions. This reliance suggests limited engagement with peer-reviewed scientific literature or evidence-based guidelines. This finding has important implications for continuing professional development, as dependence on non-peer-reviewed sources may increase variability in counseling quality and increase the risk of misinformation. Addressing this issue through structured continuing professional development, academic symposia, and integration of microbiome science into pharmacy curricula could significantly improve pharmacists’ evidence literacy and clinical confidence.

From a public health perspective, these knowledge gaps have important implications for patient care. Pharmacists, often the first point of contact for patients seeking advice on supplements, play a vital role in guiding the safe and effective use of probiotics. Strengthening their knowledge can improve counseling, support antimicrobial stewardship, and promote preventive use for conditions such as antibiotic-associated diarrhea and irritable bowel syndrome. These findings underscore the need for targeted, practice-oriented educational strategies that emphasize clinically relevant outcomes (indications, safety, patient selection, and evidence strength), rather than focusing primarily on factual recall.

### Strengths and limitations

A key strength of this study is its large and diverse sample of pharmacists from various regions, practice settings, and educational backgrounds providing a broad view of pharmacists in the Palestinian context. The study used a comprehensive questionnaire that assessed not only knowledge but also attitudes and practices, offering a multi-dimensional understanding of the topic. Furthermore, the use of both bivariable and multivariate analyses strengthens the validity of the findings.

However, several limitations should be acknowledged. Data were self-reported, which may introduce recall or social desirability bias. Because recruitment relied on online distribution and voluntary participation, selection bias and limited generalizability are possible. Given the high reported willingness to recommend probiotics, social desirability bias may have inflated favorable attitude responses. Additionally, the cross-sectional design limits the ability to establish causal relationships between variables. In addition, the survey was distributed online via email, WhatsApp, text messages, and pharmacist social media groups with snowball forwarding; therefore, the sample may over-represent pharmacists who are more digitally connected or more interested in probiotics/prebiotics, introducing potential selection bias and limiting generalizability. Because recruitment used a non-probability approach, some characteristics were unevenly distributed (e.g., a higher proportion of younger participants and community pharmacists), which may limit representativeness across all pharmacist subgroups. Although complete-case analysis was used, comparisons between included and excluded participants did not reveal significant differences in key variables, suggesting that selection bias due to missing data was likely limited.

The multivariable model demonstrated limited but statistically significant explanatory power (R² = 0.06; adjusted R² = 0.04), suggesting that measured demographic and professional characteristics explain only a small proportion of variation in knowledge score. Therefore, the observed associations should be interpreted cautiously rather than definitive. Unmeasured factors (e.g., continuing education, access to clinical resources, and workplace support) may play a larger role in shapping knowledge. Finally, the questionnaire did not include open-ended items that could have captured deeper insights into pharmacists’ reasoning or personal experiences with probiotics and prebiotics. Moreover, the study did not assess actual patient outcomes or compare findings with other health professionals, which could be explored in future ‌‌research.

## Conclusion

This study provides valuable insights into the KAP of pharmacists in Palestine regarding probiotics and prebiotics. The majority of pharmacists demonstrated moderate knowledge, and most held positive attitudes and expressed a willingness to recommend probiotics when supported by evidence. However, knowledge gaps and misconceptions remain, highlighting variability in knowledge across participants. Importantly, practice-related findings suggest variability in counseling and recommendation behaviors, highlighting the need to strengthen pharmacists’ applied clinical decision-making and confidence. To bridge these gaps, future interventions should focus on incorporating probiotic education into pharmacy curricula and continuing professional development with an emphasis on indications, safety, patient selection, and evidence appraisal. Strengthening pharmacists’ expertise in this field may enhance their confidence in recommending probiotics and ensure safer, more effective patient care.

## Supporting information

S1 File Survey on KAP regarding probiotic and prebiotic among pharmacists. (DOCX)

S1 Table Multivariable linear regression analysis of factors associated with knowledge score using robust standard errors. (DOCX)

S2 Table Responses to knowledge assessment items regarding probiotics and prebiotics among participating pharmacists. (DOCX)

S3 Table Sociodemographic Characteristics of pharmacists by knowledge score. (DOCX)

S1 Dataset De-identified participant-level dataset underlying the findings of the study (Excel format). (XLSX)
