# Supplementary material for: Pharmacists Knowledge, Attitudes, and Practices Regarding Probiotics and Prebiotics: A Cross-Sectional Study from Palestine
Source: PLoS One. 2026 Jun 18;21(6):e0350648. doi: 10.1371/journal.pone.0350648 (PMC13278477; doi:10.1371/journal.pone.0350648)
Supplement: S1 File — (DOCX) [file pone.0350648.s001.docx]

# **Factors Influencing Knowledge, Attitudes, and Practices Relating to Probiotics and Prebiotics Among Pharmacists: A Cross-Sectional Study from Palestine.**

# Section one: Sociodemographic characteristics:

| **Category** | **Options** |
| --- | --- |
| 1. Gender | ☐ Male |
|  | ☐ Female |
| 1. Age (years) | ☐ 20–29 |
|  | ☐ 30–39 |
|  | ☐ 40–49 |
|  | ☐ ≥50 |
| 1. Profile | ☐ Manager |
|  | ☐ Owner |
|  | ☐ Staff Pharmacist |
| 1. Working settings | ☐ Community pharmacies |
|  | ☐ Hospital pharmacies outpatient |
|  | ☐ Hospital pharmacies inpatient |
| 1. Educational Level | ☐ Bachelor of Pharmacy |
|  | ☐ Pharm D |
|  | ☐ Master |
|  | ☐ PhD |
| 1. Years of Experience | ☐ less than one year |
|  | ☐ 1- 5 |
|  | ☐ 5–10 |
|  | ☐ More than 10 years |
| 1. Pharmacist’s Working Hours per Week | ☐ 24 hr or less |
|  | ☐ 25–40 hr |
|  | ☐ More than 40 hr |
| 1. Geographic Location of the Pharmacy | ☐ City |
|  | ☐ Village |
|  | ☐ Camp |
| 1. Province | ☐ Al Quds |
|  | ☐ Jenin |
|  | ☐ Nablus |
|  | ☐Tulkarem |
|  | ☐ Ramallah |
|  | ☐Beitlahem |
|  | ☐ Hebron |
|  | ☐ Jericho |
|  | ☐ Tubas |
|  | ☐ Salfit |
|  | ☐Qalqilia |
| 1. Number of Hours/Week Pharmacy is Open | ☐ Less than 80 h |
|  | ☐ 80–120 h |
|  | ☐ 7 days 24/24 |
| 1. Number of handled prescriptions per day |  |
| 1. Total number of employees at the practice site |  |

# Section two: knowledge assessment

## Knowledge assessment: definition

| **What is the definition for gut microbiome?** | |
| --- | --- |
| Studies int the gut microbiota is in their infancy, there is no universal definition. | ❑ |
| The human digestive tract associated microbes (gut microbiota) are referred to as the gut microbiome (all microbial genetic material) in the gut | ❑ |
| It contains good and bad gut bacteria | ❑ |
| The human digestive tract is referred to as the gut microbiome (all microbial genetic material) in the gut | ❑ |
| **Are you familiar with the concepts of "probiotic" and "prebiotic"?** | |
| Yes, only probiotic | ❑ |
| Yes, only prebiotic | ❑ |
| Yes, I know both probiotic and prebiotic | ❑ |
| No, neither | ❑ |
| **Select the correct definitions for probiotic and prebiotic from the options below.** | |
| **Probiotics** | ❑ |
| Probiotics are live bacteria that are helpful to your health when you eat them | ❑ |
| Probiotics are fibers that help feed the good bacteria in your body | ❑ |
| Probiotics are natural antibiotics | ❑ |
| Probiotics are cleaning products to help kill bacteria on fruit and vegetables | ❑ |
| Probiotics are substances that make food taste sweeter | ❑ |
| I do not know the definition of probiotics | ❑ |
| **Prebiotics** |  |
| Prebiotics are food that you eat that can help the good bacteria in your body | ❑ |
| Prebiotics are live bacteria that are helpful to your health when you eat them | ❑ |
| Prebiotics are a type of vitamin to help health | ❑ |
| Prebiotics are drugs to help lower blood pressure | ❑ |
| Prebiotics are harmful chemicals | ❑ |
| I do not know the definition of prebiotics | ❑ |
| **Synbiotics** |  |
| A mixture of pro- and prebiotics which beneficially affects the host by improving the survival and implantation of live probiotic bacteria in the GIT, or allow the selective growth of native GI beneficial bacteria. | ❑ |
| A mixture of post- and prebiotics which beneficially affects the host by improving the survival and implantation of live probiotic bacteria in the GIT, or allow the selective growth of native GI beneficial bacteria. | ❑ |
| A mixture of pro- and fibre which beneficially affects the host by improving the survival and implantation of live probiotic bacteria in the GIT, or allow the selective growth of native GI beneficial bacteria. | ❑ |
| A chemical combination of pro- and prebiotics which beneficially affects the host by improving the survival and implantation of live probiotic bacteria in the GIT, or allow the selective growth of native GI beneficial bacteria | ❑ |
| **How would you rate your knowledge about different alternative medicine modalities?** |  |
| Limited Knowledge | ❑ |
| Moderate Knowledge | ❑ |
| Good Knowledge | ❑ |
| Extensive Knowledge | ❑ |

| **Knowledge item- probiotics** | **Correct** | **Incorrect** | **I do not know** |
| --- | --- | --- | --- |
| 1. Probiotics are live microorganisms providing a health benefit when taken in adequate amounts. |  |  |  |
| 1. Probiotics are consumed as supplements or probiotics-fortified foods. |  |  |  |
| 1. The only probiotics that work are tablets, powders, or capsules |  |  |  |
| 1. Probiotics should be taken before a meal |  |  |  |
| 1. For a beneficial effect, it is necessary to consume probiotics for a long period of time as they disappear from the gut after two weeks |  |  |  |
| 1. Probiotics could not modulate immune responses. |  |  |  |
| 1. Some probiotics products have clinically proven beneficial effects in diarrhea and lactose intolerance. |  |  |  |
| 1. Some probiotics products are effective in inflammatory bowel disease and irritable bowel syndrome. |  |  |  |
| 1. Probiotics could not play a role in urogenital conditions. |  |  |  |
| 1. Probiotics could not be effective in allergy. |  |  |  |
| 1. Probiotics are available in different forms of strains; each one has different effect(s). |  |  |  |
| 1. There are minimal risks associated with the clinical use of probiotics for most patients. |  |  |  |
| **Knowledge item- prebiotics** |  |  |  |
| 1. not degraded by human GIT acid or enzyme |  |  |  |
| 1. fermented by micrbiota |  |  |  |
| 1. confers health benefit to host |  |  |  |
| 1. selectively increases good bacteria |  |  |  |
| 1. shelf unstable |  |  |  |
| 1. Help feed the probiotics which can help build immunity |  |  |  |
| 1. Decrease absorption of calcium and magnesium |  |  |  |
| 1. reduced triglyceride in hypercholesterolemia |  |  |  |

## Knowledge of Microbial Species including Probiotic Strains

| **Select species from the below list of microorganisms that you believe contain probiotic strains.** | |
| --- | --- |
| Lactobacillus acidophilus | ❑ |
| Bifidobacterium bifidum | ❑ |
| Mycobacterium avium | ❑ |
| Escherichia coli | ❑ |
| Lactobacillus rhamnosus | ❑ |
| Bacillus subtilis | ❑ |
| Enterococcus faecium | ❑ |
| Saccharomyces boulardii | ❑ |

| **Select species from the below list of microorganisms that you believe contain prebiotic., food source** | |
| --- | --- |
| Natural or synthetics |  |
| Galacto-oligosaccharides | ❑ |
| Inulin-type fructans | ❑ |
| Fructo-oligosaccharides | ❑ |
| Onions |  |
| Potato chips |  |
| Garlic |  |
| Sunchokes |  |

# Section three: Practice

| Practice items | Never | Rarely | Sometimes | Often | Always |
| --- | --- | --- | --- | --- | --- |
| How often do you give nutritional advice in your practice? |  |  |  |  |  |
| How often do you advise probiotics in your practice? |  |  |  |  |  |

# Section four: Reason to/not advice on probiotics

| 1. **Have you ever recommended that your patients use probiotics?** | |
| --- | --- |
| Yes move to question 2,3 | ❑ |
| No move to question 4 | ❑ |
| 1. **Reasons for Advising using probiotic/ prebiotics** |  |
| Patients request | ❑ |
| No harm no foul | ❑ |
| insufficient efficacy of other therapies | ❑ |
| Following of colleagues’ advice | ❑ |
| Good experience with probiotics | ❑ |
| Adherence to guideline | ❑ |
| No harm no foul | ❑ |
| Evidence on probiotic efficacy | ❑ |
| As part of healthy lifestyle | ❑ |
| 1. **Indication for probiotic advice** |  |
| for a patient During and/or after a course of antibiotics | ❑ |
| For IBS symptoms | ❑ |
| For diarrhoea | ❑ |
| If a patient is run down and/or susceptible to common infections | ❑ |
| Constipation | ❑ |
| Diverticulitis | ❑ |
| Ulcerative colitis | ❑ |
| Before travel abroad | ❑ |
| Older people | ❑ |
| For mothers or babies in families with tendency to allergy | ❑ |
| for improved digestion | ❑ |
| to reduce bloating | ❑ |
| Dermatological problems | ❑ |
| Dental health | ❑ |
| Stress | ❑ |
| 1. **Indication for prebiotic advice** |  |
|  |  |
|  |  |
|  |  |
|  |  |
| 1. **Reason for not recommending probiotics/ prebiotics** |  |
| Little or no idea about probiotics. | ❑ |
| There are no clinical applications for probiotics in my specialty | ❑ |
| Not convinced of clinical benefit | ❑ |
| Cost | ❑ |
| Negative experiences with prior use | ❑ |
| Lack of knowledge about Clinical use of Probiotics | ❑ |
| Lack of information regarding available probiotics products | ❑ |
| Limited or non-availability of clinically proven probiotics products | ❑ |
| Clinical use of probiotics is controversial. | ❑ |
| Doubt in the quality of probiotics products | ❑ |
| No data on safety of probiotics. | ❑ |
| Dietary supplements, like probiotics, are not regulated by the FDA | ❑ |
| Traditional yogurts are as effective as probiotics products. | ❑ |
| The efficacy of probiotics is inferior to, or does not provide additional benefit over standard therapeutics | ❑ |
| Negative experience with prior use of probiotics | ❑ |
| Risk of infection due to probiotics‘ use | ❑ |

# Section five: Attitude about Probiotics

| **What are your views about Probiotics?** | **Not at all** | **Some what** | **Very much** |
| --- | --- | --- | --- |
| Do you consider probiotics are good for human |  |  |  |
| Do you consider that probiotics can be dangerous for health? |  |  |  |
| If we provide you proper reference about probiotics use, would you be willing to recommend probiotics to your patients? |  |  |  |

# Section six: Source of information

| **Have you ever received any specific education on Probiotic/ prebiotics?** | | | |
| --- | --- | --- | --- |
| Yes | ❑ | | |
| No | ❑ | | |
| Not sure | ❑ | | |
| **Through which of the following have you received education regarding probiotic and prebiotic? (You may choose more than one answer)** | | | |
| Internet (general) | ❑ | | |
| Book | ❑ | | |
| Scholarly articles | ❑ | | |
| Radio or TV | ❑ | | |
| Distributed material through my department | ❑ | | |
| Mandatory internet training modules through Graduate Medical Education | ❑ | | |
| Discussion with co-workers | ❑ | | |
| Grand rounds lecture by member of my department | ❑ | | |
| Other (please specify) | __________________ | | |
| **Are you interested in learning more about probiotic/ prebiotics?** | | | |
| Yes | ❑ | | |
| No | ❑ | | |
| **Preferred source of future information** | | |  |
| E-learning | | ❑ |  |
| Scientific journals | | ❑ |  |
| Social media | | ❑ |  |
| Lay journal (public) | | ❑ |  |
| Symposia | | ❑ |  |
| Email/ Newsletter | | ❑ |  |
| No more information | | ❑ |  |
| Others (please specify) | | ❑ |  |
| **Preferred Type of Future Information** | | |  |
| Efficacy | | ❑ |  |
| Safety | | ❑ |  |
| Mode of action | | ❑ |  |
| Colleagues experiences | | ❑ |  |
| Cost | | ❑ |  |
| No more information is needed | | ❑ |  |
| Other (please specify) | | ❑ |  |
