# Supplementary material for: Pharmacists Knowledge, Attitudes, and Practices Regarding Probiotics and Prebiotics: A Cross-Sectional Study from Palestine
Source: PLoS One. 2026 Jun 18;21(6):e0350648. doi: 10.1371/journal.pone.0350648 (PMC13278477; doi:10.1371/journal.pone.0350648)
Supplement: S1 Table — (DOCX) [file pone.0350648.s002.docx]

S1Table. Multivariable linear regression analysis of factors associated with knowledge score using robust standard errors (HC1)

| **Variable** | **Category** | **B (Robust SE)** | **p-value** |
| --- | --- | --- | --- |
| **Probiotic education** | Yes vs No | 2.00 (0.64) | 0.002 |
| **Age (years)** | 20–29 vs ≥50 | 4.21 (1.76) | 0.018 |
|  | 30–39 vs ≥50 | 4.72 (1.89) | 0.013 |
|  | 40–49 vs ≥50 | 5.48 (2.21) | 0.014 |
| **Gender** | Male vs Female | -0.50 (0.72) | 0.487 |
| **Prescriptions per day** | <50 vs ≥50 | 1.68 (0.72) | 0.021 |
| **Practice setting** | Hospital vs Community | -0.91 (1.17) | 0.435 |

**Notes:** B = unstandardized regression coefficient; SE = standard error (robust, HC1). Reference categories: No probiotic education, age ≥50 years, female gender, ≥50 prescriptions/day, community pharmacy setting. Robust standard errors (HC1) were used to account for potential heteroscedasticity. Results were consistent with the primary model.
