# Supplementary material for: Pharmacists Knowledge, Attitudes, and Practices Regarding Probiotics and Prebiotics: A Cross-Sectional Study from Palestine
Source: PLoS One. 2026 Jun 18;21(6):e0350648. doi: 10.1371/journal.pone.0350648 (PMC13278477; doi:10.1371/journal.pone.0350648)
Supplement: S2 Table — (DOCX) [file pone.0350648.s003.docx]

S2 Table: Responses to knowledge assessment items regarding probiotics and prebiotics among participating pharmacists

| Indicator | N | % |
| --- | --- | --- |
| 4- What is the definition for gut microbiome ? | 258 | 65% |
| Probiotics" | 355 | 89% |
| 7- Select the correct definitions for probiotic and prebiotic from the options below: Prebiotics | 268 | 67% |
| 8- Select the correct definitions for probiotic and prebiotic from the options below:  Synbiotics | 324 | 81% |
| 9- select wether we can use probiotics for these certain indications or not . ( select Yes or No ) [Boost the human immune system] | 249 | 62% |
| 9- select wether we can use probiotics for these certain indications or not .  ( select Yes or No ) [Treat gastrointestinal disorders (GERD,IBS..ect)] | 361 | 90% |
| 9- select wether we can use probiotics for these certain indications or not . ( select Yes or No ) [Reduce the reccurance of urinary tract infections] | 92 | 23% |
| 9- select wether we can use probiotics for these certain indications or not .  ( select Yes or No ) [As alternative for antibiotics] | 305 | 76% |
| 9- select wether we can use probiotics for these certain indications or not .  ( select Yes or No ) [Alleviate depression symptoms] | 191 | 48% |
| 9- select wether we can use probiotics for these certain indications or not .  ( select Yes or No ) [Improve overall cardiovascular system] | 186 | 47% |
| 9- select wether we can use probiotics for these certain indications or not .  ( select Yes or No ) [Resolve allergy symptoms] | 175 | 44% |
| 9- select wether we can use probiotics for these certain indications or not .  ( select Yes or No ) [Improve respiratoory tract immunity] | 182 | 46% |
| 9- select wether we can use probiotics for these certain indications or not . ( select Yes or No ) [Improve oral health] | 318 | 80% |
| 9- select wether we can use probiotics for these certain indications or not .  ( select Yes or No ) [Overall vaginal health] | 300 | 75% |
| 11- knowledge item- probiotic [Probiotics are live microorganisms providing a health benefit when taken in adequate amounts.] | 331 | 83% |
| 11- knowledge item- probiotic [Probiotics are consumed as supplements or probiotics-fortified foods.] | 300 | 75% |
| 11- knowledge item- probiotic [The only probiotics that work are tablets, powders, or capsules] | 200 | 50% |
| 11- knowledge item- probiotic [Probiotics should be taken before a meal] | 194 | 49% |
| 11- knowledge item- probiotic [For a beneficial effect, it is necessary to consume probiotics for a long period of time as they disappear from the gut after two weeks] | 178 | 45% |
| 11- knowledge item- probiotic [Probiotics could not modulate immune responses] | 213 | 53% |
| 11- knowledge item- probiotic [Some probiotics products have clinically proven beneficial effects in diarrhea and lactose intolerance.] | 289 | 72% |
| 11- knowledge item- probiotic [Some probiotics products are effective in inflammatory bowel disease and irritable bowel syndrome.] | 303 | 76% |
| 11- knowledge item- probiotic [Probiotics could not play a role in urogenital conditions.] | 186 | 47% |
| 11- knowledge item- probiotic [Probiotics could not be effective in allergy.] | 133 | 33% |
| 11- knowledge item- probiotic [Probiotics are available in different forms of strains; each one has different effect(s)] | 248 | 62% |
| 11- knowledge item- probiotic [There are minimal risks associated with the clinical use of probiotics for most patients.] | 173 | 43% |
| 12- Knowledge item- prebiotics [not degraded by human GIT acid or enzyme] | 168 | 42% |
| 12- Knowledge item- prebiotics [fermented by micrbiota] | 213 | 53% |
| 12- Knowledge item- prebiotics [confers health benefit to host] | 285 | 71% |
| 12- Knowledge item- prebiotics [selectively increases good bacteria] | 275 | 69% |
| 12- Knowledge item- prebiotics [Shelf unstable ] | 78 | 20% |
| 12- Knowledge item- prebiotics [Help feed the probiotics which can help build immunity] | 266 | 67% |
| 12- Knowledge item- prebiotics [Decrease absorption of calcium and magnesium] | 62 | 16% |
| 12- Knowledge item- prebiotics [reduced triglyceride in hypercholesterolemia] | 128 | 32% |
| 13- Select species from the below list of microorganisms that you believe contain probiotic strains. | 390 | 98% |
| 14- Select species from the below list of microorganisms that you believe contain prebiotic., food source.   (You may choose more than one answer) | 332 | 83% |
