# Supplementary material for: Pharmacists Knowledge, Attitudes, and Practices Regarding Probiotics and Prebiotics: A Cross-Sectional Study from Palestine
Source: PLoS One. 2026 Jun 18;21(6):e0350648. doi: 10.1371/journal.pone.0350648 (PMC13278477; doi:10.1371/journal.pone.0350648)
Supplement: S3 Table — (DOCX) [file pone.0350648.s004.docx]

S3 Table: Sociodemographic Characteristics of pharmacists by knowledge score (n=400)

| **Category** | **Total n=400 n(%)** | **Poor Knowledge n(%)** | **Moderate Knowledge n(%)** | **High Knowledge n(%)** | **P-value** |
| --- | --- | --- | --- | --- | --- |
| **Gender** |  |  |  |  | 0.197 |
| Male | 135 (33.7) | 19 (14.1%) | 75 (55.6%) | 41 (30.4%) |  |
| Female | 265 (66.3) | 22 (8.3%) | 156 (58.9%) | 87 (32.8%) |  |
| **Age (years)** |  |  |  |  | 0.297 |
| 20-29 | 297 (74) | 30 (10.1%) | 172 (57.9%) | 95 (32.0%) |  |
| 30-39 | 63 (15.6) | 5 (7.9%) | 39 (61.9%) | 19 (30.2%) |  |
| 40-49 | 25 (6.3) | 3 (12.0%) | 10 (40.0%) | 12 (48.0%) |  |
| ≥50 | 15 (3.8) | 3 (20.0%) | 10 (66.7%) | 2 (13.3%) |  |
| **Profile** |  |  |  |  | 0.676 |
| Manager | 28 (7) | 5 (17.9%) | 14 (50.0%) | 9 (32.1%) |  |
| Owner | 42 (10.5) | 4 (9.5%) | 23 (54.8%) | 15 (35.7%) |  |
| Staff Pharmacist | 330 (82.5) | 32 (9.7%) | 194 (58.8%) | 104 (31.5%) |  |
| **Working Settings** | |  |  |  | 0.879 |
| Community pharmacies | 369 (92.3) | 38 (10.3%) | 212 (57.5%) | 119 (32.2%) |  |
| Hospital pharmacies Outpatient | 11 (2.8) | 1 (9.1%) | 8 (72.7%) | 2 (18.2%) |  |
| Hospital pharmacies inpatient | 20 (5) | 2 (10.0%) | 11 (55.0%) | 7 (35.0%) |  |
| **Educational Level** | |  |  |  | 0.531 |
| Bachelor of Pharmacy | 327 (81.8) | 32 (9.8%) | 194 (59.3%) | 101 (30.9%) |  |
| Pharm D | 35 (8.8) | 3 (8.6%) | 20 (57.1%) | 12 (34.3%) |  |
| Master | 31 (7.8) | 4 (12.9%) | 14 (45.2%) | 13 (41.9%) |  |
| PhD | 7 (1.8) | 2 (28.6%) | 3 (42.9%) | 2 (28.6%) |  |
| **Years of Experience** | |  |  |  | 0.073 |
| less than one year | 100 (25) | 10 (10.0%) | 59 (59.0%) | 31 (31.0%) |  |
| 1-5 years | 240 (60) | 24 (10.0%) | 144 (60.0%) | 72 (30.0%) |  |
| >5–10 | 16 (4) | 0 (0.0%) | 12 (75.0%) | 4 (25.0%) |  |
| More than 10 years | 44 (11) | 7 (15.9%) | 16 (36.4%) | 21 (47.7%) |  |
| **Working Hours/Week** | |  |  |  | 0.12 |
| 24 hrs or less | 129 (32.3) | 19 (14.7%) | 76 (58.9%) | 34 (26.4%) |  |
| 25–40 hrs | 170 (42.5) | 11 (6.5%) | 101 (59.4%) | 58 (34.1%) |  |
| More than 40 hrs | 101(25.3) | 11 (10.9%) | 54 (53.5%) | 36 (35.6%) |  |
| **Residency** | |  |  |  | 0.172 |
| City | 262 (65.5) | 32 (12.2%) | 146 (55.7%) | 84 (32.1%) |  |
| Village | 122 (30.5) | 6 (4.9%) | 76 (62.3%) | 40 (32.8%) |  |
| Camp | 16 (4) | 3 (18.8%) | 9 (56.3%) | 4 (25.0%) |  |
| **Pharmacy Open Hours/Week** | | | |  | 0.551 |
| Less than 80 h | 74 | 10 (13.5%) | 39 (52.7%) | 25 (33.8%) |  |
| 80–120 h | 263 | 25 (9.5%) | 159 (60.5%) | 79 (30.0%) |  |
| 7 days 24/24 | 63 | 6 (9.5%) | 33 (52.4%) | 24 (38.1%) |  |
| **Prescriptions/Day** | |  |  |  | 0.044* |
| less than 50 | 245 | 14 (5.7%) | 146 (59.6%) | 85 (34.7%) |  |
| 50 and more | 103 | 14 (13.6%) | 54 (52.4%) | 35 (34.0%) |  |
| **Employees at Site** | |  |  |  | 0.791 |
| < 5 | 299 | 27 (9.0%) | 170 (56.9%) | 102 (34.1%) |  |
| ≥5 | 48 | 3 (6.3%) | 29 (60.4%) | 16 (33.3%) |  |
| **Have you ever used probiotic for yourself** | | | | | 0.154 |
| Yes | 161 (40) | 11(6.8%) | 94(58.4%) | 56 (34.8%) |  |
| No | 239 (60) | 30(12.6) | 137(57.3%) | 72 (30.1%) |  |
| **Have you ever received any specific education on Probiotic/ prebiotics?** | | | | | < 0.001 |
| Yes | 104 (26.0) | 21 (20.2%) | 58 (55.8%) | 25 (24.0%) |  |
| No | 228 (57.0) | 13 (5.7%) | 125 (54.8%) | 90 (39.5%) |  |
| Unsure | 68 (17.0) | 7 (10.3%) | 48 (70.6%) | 13 (19.1%) |  |
