# Supplementary material for: Pharmacists Knowledge, Attitudes, and Practices Regarding Probiotics and Prebiotics: A Cross-Sectional Study from Palestine
Source: PLoS One. 2026 Jun 18;21(6):e0350648. doi: 10.1371/journal.pone.0350648 (PMC13278477; doi:10.1371/journal.pone.0350648)
Supplement: Attachment — Submitted filename: Reviewer report- V2.docx [file pone.0350648.s008.docx]

# Date: 2 Mar. 26

# Re: Response to reviewers’ comments on the manuscript:

# “Pharmacists Knowledge, Attitudes, and Practices Regarding Probiotics and Prebiotics: A Cross-Sectional Study from Palestine”.

# The authors would like to thank the Editor for considering the manuscript for publication in PLOS ONE. The authors would also like to thank the Reviewers for their time and constructive comments and suggestions to improve the quality of the manuscript. The following responses have been prepared to address the comments in a point-by-point fashion.

# Reviewers' comments:

# Reviewer #1:

## Abstract

## 1. The study objectives are described inconsistently throughout the manuscript. For example:

“This study aimed to assess pharmacists’ KAP related to probiotics and prebiotics and to identify demographic and professional factors influencing knowledge.”

“Therefore, this study aimed to assess the knowledge, attitudes, and practices of pharmacists in Palestine regarding probiotics and prebiotics, and to identify factors that may influence these domains.”

“The statistical analysis employed included both descriptive and inferential statistics to provide a comprehensive understanding of the factors influencing pharmacists professionals' knowledge and attitudes toward probiotics and prebiotics.”

The authors should ensure that the study objective is clearly defined and consistently stated throughout the manuscript.

**Response:** We thank the reviewer for this constructive comment.

**Revised standardized objective** (now used consistently):

“To assess pharmacists’ knowledge, attitudes, and practices (KAP) regarding probiotics and prebiotics in Palestine, and to identify demographic and professional factors associated with pharmacists’ knowledge score.”

**Where revised:**

Abstract: Aim sentence revised to match the standardized objective exactly.

Introduction (last paragraph): Objective sentence revised to match verbatim.

Methods/Statistical Analysis: Removed wording implying analysis of “factors influencing attitudes” and clarified that multivariable analysis examined factors associated with knowledge score (with attitudes and practices summarized descriptively).

## 2. “The mean knowledge score was 21.3 ± 6.1 (range: 4-34). Most participants demonstrated moderate knowledge (57.8%), while 32.0% achieved high knowledge and 10.3% had poor knowledge. Attitudes were generally positive, with 85.5% believed in their health benefits and 81% were willing to recommend them. In multivariable analysis, years of experience was independently associated with knowledge score (p= 0.027). Practice findings indicated variable self-reported counseling and recommendation behaviors, with barriers related to evidence access and confidence in clinical application.”

In the Abstract, the results are not presented in a fully consistent KAP sequence. The findings should be reported systematically in the following order: knowledge, attitudes, and then practices. A similar structural issue is observed in the Discussion section, where the interpretation of results should also follow the same logical order for clarity and coherence.

**Response:** Thank you for this helpful suggestion. For clarity and coherence, we have restructured both sections (Results- Abstract and discussion) to present and interpret findings systematically in the KAP order.

## Methods

## 3. “Because recruitent relied on online distribution and voluntary participation, selection bias and limited generalizability are possible.”

This reflects a study limitation and should therefore be moved to the Discussion section under the limitations subsection, rather than appearing in the Methods.

**Response:** Thank you for this observation. We agree that the statement regarding selection bias and generalizability is a study limitation and should not appear in the Methods section.

**Change made:** We deleted this sentence from the Methods and moved it to the Discussion under the Limitations subsection.

## 4. In the Statistical Analysis section, the authors do not describe the use of univariable and multivariable regression models, nor do they explain the criteria for variable selection in the multivariable model (e.g., p-value threshold, theoretical relevance, stepwise approach, etc.). These methodological details are essential for transparency and reproducibility and should be clearly reported.

**Response:** Thank you for this comment. We revised the Statistical Analysis section to explicitly report univariable and multivariable regression models

## Results

## 5. “The majority of participants were female (66.3%), aged between 20 and 29 (74.3%)”

“With 57% reported no formal training, 28.5% indicated they had received some education, and 17% were unsure of their knowledge.”

The authors should ensure that the numerical data presented in the text are fully consistent with those reported in the corresponding tables.

**Response:** Thank you for pointing this out. We agree that all numerical values reported in the narrative must match the corresponding tables exactly to avoid confusion and to ensure internal consistency.

**Change made:** We re-checked all demographic and training-related figures in the Results text against the values in Table 1 (and other relevant tables) and corrected any discrepancies. Specifically, we ensured that the reported proportions for sex, age group (20–29 years), and formal training/education regarding probiotics and prebiotics are now identical in both the text and the tables. Where needed, we also standardized rounding and decimal places to match the tables.

## 6. Several rows in Table 1 do not include percentages. Additionally, the number of decimal places is not consistent across the manuscript. The authors should standardize the reporting format throughout the paper.

**Response:** Agreed. Presentation consistency matters.

**Change made:** Added missing percentages to Table 1 rows where counts were provided without percentages.

Standardized percent formatting across tables (consistent decimal places throughout).

## 7. In Table 1, for the variable “Pharmacy open hours/week,” the category structure appears incomplete. There seems to be a missing category between >120 hours and <7 days. The authors should review and clarify the categorization.

**Response:** We understand the concern. The intention was to represent three operational patterns (<80, 80–120, and 24/7). The third category corresponds to >120 hours/week.

**Change made:** We revised the label to an unambiguous form:

“24/7 (7 days × 24 h/day; >120 h/week)”

This makes the implied weekly-hours meaning explicit and removes the appearance of a missing interval.

## 8. In Table 2, the response categories should be arranged in descending order of percentage to improve readability and facilitate interpretation.

**Response:** Thank you for this comment. Agreed.

**Change made:** Table 2 response categories were reordered from highest to lowest percentage to improve interpretability.

## 9. In Table 3, the total number of responses in at least one row does not equal 400. The authors should verify and correct the calculations to ensure internal consistency.

**Response:** Thank you for noting this issue. We agree that the original Table 3 contained an internal inconsistency in which the total number of responses for some knowledge items did not equal the study sample size (N = 400). This occurred due to an error in how the table was compiled, which led to incorrect row totals.

**Change made:** We have corrected Table 3 by re-generating the frequency table directly from the final dataset using the full sample as the denominator (N = 400) and ensuring that each knowledge item has mutually exclusive response categories (Correct / Incorrect / Not sure) that sum to 400. Percentages were recalculated accordingly as n/400 × 100. The revised table now shows consistent totals (400) for every row and internally consistent percentages.

## 10. Regarding Table 5:

## The reported regression results indicate a very low explanatory power (R² = 0.034), and more importantly, a negative adjusted R² (Adjusted R² = −0.002). A negative adjusted R² suggests that, after accounting for the number of predictors in the model, the model performs worse than an intercept-only model. In other words, the included independent variables do not meaningfully improve the prediction of the dependent variable. This raises concerns regarding the adequacy of the model specification and the theoretical relevance of the selected predictors. The authors are encouraged to re-examine the model, justify the inclusion of the predictors, and discuss the implications of this extremely low explanatory power. At minimum, this limitation should be explicitly acknowledged in the manuscript.

## In addition, the structure of Table 5 is unclear. For categorical variables, one category is typically selected as the reference group, and regression coefficients are presented for the remaining categories. However, in this table, each categorical variable appears to be represented by only one row. For example, in Table 1, education level is divided into four categories (Bachelor, PharmD, Master, and PhD), yet in Table 5, only a single row is shown for this variable. The authors should clarify how categorical variables were coded and analyzed in the regression model.

**Response:** Thank you for this important comment. We agree that the model has very limited explanatory power (R² = 0.034; adjusted R² = −0.002), and we have explicitly acknowledged this limitation in the manuscript. We also agree that the original Table 5 presentation did not clearly explain how categorical predictors were handled, which could lead to misinterpretation.

Clarification (categorical coding): In the regression model, several categorical variables (e.g., education level, province, profile, working setting, and geographic location) were entered into SPSS using their numeric coding as single predictors rather than being expanded into indicator (dummy) variables with an explicit reference category. Therefore, the coefficients shown for these variables represent the expected change in knowledge score per one-unit increase in the coding scale, and do not represent category-versus-reference comparisons.

**Change made (Table 5 and Methods):** To ensure transparency and reproducibility, we revised the Statistical Analysis section and added a clear note to Table 5 stating that these categorical predictors were analyzed as coded variables and explaining how the coefficients should be interpreted. We also corrected the Durbin–Watson value (1.896) and clarified that the multivariable regression was conducted using complete cases (n = 332, based on the ANOVA degrees of freedom).

**Change made (Discussion- Limitations):** We added a limitation statement noting that the model explains only a small proportion of variance in knowledge score and that unmeasured factors (e.g., continuing education exposure and access to evidence-based resources) likely play a larger role.

## 11. There are still numerous typographical errors and awkward expressions throughout the manuscript. A thorough language revision by a professional English editor is strongly recommended.

**Response:** Thank you for this comment. We agree that the manuscript required language polishing to improve clarity and readability.

**Change made:** We performed a comprehensive language revision across the entire manuscript to correct typographical and grammatical errors, improve sentence structure, and resolve awkward phrasing. We also standardized terminology and formatting (e.g., consistent use of KAP terminology, punctuation, and decimal reporting) to enhance overall readability.

Reviewer #4: This is a good study regarding KAP, and helps to inform improvement of pharmacists practice regarding probiotics and prebiotics. Here are my comments:

1. Knowledge score of 50% fall in poor and fair & 70% in fair & knowledgeable as mention in abstract and statistical analysis - 1. Poor Knowledge: 50% or less (≤ 12 points); 2. Fair Knowledge: Scores between 50% and 70% (13-24 points); 3. Knowledgeable: Scores of 70% or higher (≥ 25 points). It should have clear cut off point between these scores.

**Response:** Thank you for this comment. We would like to clarify that the knowledge cutoffs used in our study are already mutually exclusive and do not overlap at 50% or 70%. However, we agree that the wording in the manuscript could be interpreted ambiguously.

1. Poor Knowledge: ≤ 50% (≤ 17 points)
2. Moderate Knowledge: Scores between >50% and <70% (18-23 points)
3. High Knowledge: Scores of ≥70% ( 24-34 points)

## 2. As this is cross sectional study please consider include reporting findings accordance to STROBE in study design.

**Response:** Thank you for this recommendation. We agree that STROBE is the appropriate reporting guideline for cross-sectional studies and improves completeness and transparency.

**Change made:** We reviewed the manuscript against the STROBE checklist for cross-sectional studies and revised the text to ensure key items are explicitly reported, including: study design and setting, eligibility criteria and recruitment, variables and measurement, efforts to address potential bias, sample size rationale, handling of missing data, and detailed statistical methods.

## 3. “Questionnaire was reviewed by a panel of specialists in the fields of pharmacy”- Please indicate how many panels were involved for this validity.

**Response:** Clarification accepted. The panel consisted of four experts.

**Change made:** We explicitly stated the panel size and composition (4 members)

## 4. Was the pilot study conducted online to 20 respondents? Please state in procedure

**Response:** Thank you for this clarification request. The pilot study was conducted by approaching pharmacists in person, and participants completed the draft questionnaire electronically using Google Forms to assess clarity, comprehension, and feasibility.

**Change made:** We revised the Survey design and validation section to explicitly state the pilot administration method and to clarify that pilot responses (n = 20) were excluded from the final analysis and not included in the final study sample.

“Afterward, a pilot study (n = 20) was conducted by approaching pharmacists in person. Participants completed the draft questionnaire electronically using Google Forms to assess clarity, comprehension, and completion time. Feedback resulted in minor refinements, and pilot responses were excluded from the final dataset and analyses.”

## 5. Data analysis part- Is there any missing data and how authors handled missing data from respondents?

**Response:** Thank you for raising this important point. Yes, there were some missing responses for selected variables (e.g., prescription volume and number of employees), as indicated in Table 1. For descriptive analyses, we reported frequencies and percentages and explicitly presented missing categories where applicable. For inferential analyses, we used complete-case (listwise) analysis: participants with missing values in any variable included in the regression model were excluded from that model.

**Change made:** We have revised the Methods (Statistical analysis) to clearly describe the handling of missing data

## 6. Results – Please include mean,SD of respondents age for better clarity of demographic respondents.

**Response:** Thank you for the suggestion. In this survey, age was collected as categorical ranges (20–29, 30–39, 40–49, ≥50) rather than as a continuous variable; therefore, an exact mean and SD cannot be computed from the raw data.

## 7. Please do not repeat the result values in discussion section. E.g (57.8%), 32.0%, 10.3%

**Response:** Thank you for this suggestion. We agree that the Discussion should primarily interpret findings rather than restate detailed numerical results that are already presented in the Results section.

**Change made:** We revised the Discussion to remove repeated reporting of specific percentages (e.g., “57.8%, 32.0%, 10.3%”) and instead focused on interpretation of the overall pattern of findings (e.g., predominance of moderate knowledge, generally positive attitudes, and variability in practices). Where numerical values were necessary for emphasis, we retained only a minimal number of key figures and referred readers to the relevant tables for details.
