# Supplementary material for: Pharmacists Knowledge, Attitudes, and Practices Regarding Probiotics and Prebiotics: A Cross-Sectional Study from Palestine
Source: PLoS One. 2026 Jun 18;21(6):e0350648. doi: 10.1371/journal.pone.0350648 (PMC13278477; doi:10.1371/journal.pone.0350648)
Supplement: Attachment — Submitted filename: Reviewer Report-DD.docx [file pone.0350648.s007.docx]

# Date: 23^rd^ January 2026

# Re: Response to reviewers’ comments on the manuscript:

“Pharmacists Knowledge, Attitudes, and Practices Regarding Probiotics and Prebiotics: A Cross-Sectional Study from Palestine”.

The authors would like to thank the Editor for considering the manuscript for publication in PLOS ONE. The authors would also like to thank the Reviewers for their time and constructive comments and suggestions to improve the quality of the manuscript. The following responses have been prepared to address the comments in a point-by-point fashion.

# Reviewers' comments:

# Reviewer #1:

**Abstract**

## “Knowledge was assessed using a scoring system, participants were categorized as having poor (≤50), fair (50–70%), or good (≥70%) knowledge.” Is 50% considered poor or fair? Is 70% considered fair or good?

**Response:** Correct—this was ambiguous due to overlapping boundaries. In Methods we define the cutoffs explicitly (≤50%, 50–70%, ≥70%), but the Abstract wording caused overlap.

**Change:** Abstract revised to use non-overlapping thresholds:

“poor (≤50%; 0–17 points), moderate (>50% to <70%; 18–23 points), or high (≥70%; 24–34 points)”

# Introduction

## “Typically found in foods like chicory root, garlic, and onions, which promote respthe growth of beneficial gut bacteria [7].” This is not a complete sentence.

**Response:** Thank you for noting this. We agree the sentence was a fragment and also contained a typographical error (“respthe”). We revised the wording to form a complete sentence and corrected the typo while maintaining the intended meaning and citation.

**Change:** Revision made in manuscript:

“Prebiotics are non-digestible substrates that are selectively utilized by host microorganisms and may confer health benefits; they are naturally present in foods such as chicory root, garlic, and onions, which can promote the growth of beneficial gut bacteria [7]”.

# Methods

## “After conducting a comprehensive literature review, a self-administered questionnaire was created (Supplementary file 1).” sIt is unclear where the authors’ Supplementary file is located.

**Response:** We agree and have clarified this. We have uploaded the questionnaire as Supporting Information and revised the Methods text to explicitly indicate its location and label in the manuscript. The questionnaire is now provided as Supplementary file 1 and is referenced consistently throughout the manuscript.

# Results

## In Table 1, please pay attention to the groups of each variable. For example, for age, the authors have 5 groups, but age 50 is missing. For years of experience, the authors have 2 groups: 1–5 and 5–10; where should those with exactly 5 years of experience be placed? The variable "Employees at site" also misses the value

**Response:** Thank you for pointing this boundary-definition errors. We revised Table 1 to ensure all categories are mutually exclusive and collectively exhaustive, and we clarified category labels to explicitly include boundary values.

## “Associations of knowledge score and sociodemographic variables and” And what?

**Response:** We agree and corrected the truncated subtitle.

**Change**: Revised the subtitle to: “Associations between knowledge score and sociodemographic and professional characteristics.”

## Table 4 presents the adjusted chi-square analysis of knowledge levels among pharmacists who reported using probiotics.

This section has been modified, and the results are now presented in Table 5.

## It is unclear which Table 4 is being referred to.

**Response:** We agree. This was due to an incorrect table numbering/cross-reference in the Results section. We have corrected the table numbering throughout the manuscript and updated the in-text citation to refer to the correct table. All table callouts and captions were verified to ensure consistency.

# Discussion

## I see the authors aimed to evaluate the KAP of pharmacists about probiotics and prebiotics, but the practice section is not emphasized. The authors do not discuss this part, nor mention practice in the Abstract or Conclusions.

**Response:** Thank you for this important observation. We agree that the initial version under-emphasized the practice component. We have revised the manuscript to explicitly include practice in the Abstract and Conclusion, and expand the Discussion to address pharmacists’ self-reported counseling/recommendation practices and barriers, linking these findings to practice-oriented educational needs.

## “A key strength of this study is its large and diverse sample of pharmacists from various regions, practice settings, and educational backgrounds allowing for a representative view in Palestinian context.” Please review this sentence and add the limitations of convenience sampling. Since the authors collected samples online, many variables are unevenly distributed among groups. For example, age is concentrated in younger groups, mostly community pharmacists, and work experience is generally low.

**Response:** Thank you for this comment. We agree that the original wording overstated representativeness. We revised the strength statement and explicitly added the limitations related to online non-probability recruitment (convenience sampling with snowball dissemination), including potential selection bias, uneven distribution of participant characteristics (predominance of younger pharmacists and community pharmacy respondents), and generally lower work experience, which may limit generalizability.

## “All relevant data are within the manuscript and its Supporting Information files.” “The datasets used and/or analyzed during the current study are available from the corresponding author on reasonable request.” Please double-check PLOS ONE’s policy on sharing raw data.

**Response:** Thank you for raising this.

**Change:** De-identified participant-level data underlying the findings are provided as Supporting Information (S1 Dataset; Excel format). Summary data are included in the manuscript and Supporting Information. The dataset has been anonymized to protect participant privacy.

## There are many spelling errors. Please check the manuscript.

**Response:** Thank you for pointing this out. We performed a comprehensive language edit of the entire manuscript to correct spelling, grammar, punctuation, and phrasing. We also checked consistency of terminology and removed duplicated or awkward wording to improve readability.

# Reviewer #2:

The manuscript addresses a timely and relevant topic, and the overall cross-sectional design is appropriate for the stated aims. The data generally support the conclusion that pharmacists demonstrate moderate knowledge and positive attitudes toward probiotics and prebiotics. However, several methodological and conceptual issues limit the technical rigor of the study and weaken the strength of the conclusions.

# Introduction:

## The discussion of the therapeutic potential of probiotics (e.g., via the gut–brain axis) is overly brief and lacks sufficient critical context. This section would benefit from expansion using key references and a clearer rationale for why pharmacists—rather than, or in addition to, dietitians and gastroenterologists—require this knowledge. Explicitly acknowledging the primary role of dietitians in nutritional counseling would strengthen the justification for why pharmacists’ knowledge may be comparatively limited yet still clinically important given their accessibility and frequent role in over-the-counter (OTC) recommendations.

**Response:** Thank you for this valuable suggestion. We agree that the Introduction required stronger scientific context and a clearer rationale for pharmacists’ relevance. We expanded the Introduction to (1) define probiotics and prebiotics using widely accepted consensus definitions, (2) briefly contextualize the microbiota–gut–brain axis and emphasize that evidence is evolving and often strain/indication-specific, (3) acknowledge that dietitians are primary providers of nutrition counseling, and (4) justify pharmacists’ importance because probiotics/prebiotics are frequently obtained over-the-counter and pharmacists are highly accessible healthcare professionals who counsel on supplements and self-care products.

**Change:** revision made to manuscript;

- Expanded the Introduction to include consensus definitions of probiotics/prebiotics/synbiotics and to add concise context on the microbiota–gut–brain axis with an evidence-cautious framing.
- Added explicit acknowledgement that dietitians are primary providers of nutrition counseling and that gastroenterologists often lead gastrointestinal clinical management.
- Added a clear rationale for pharmacists’ relevance by highlighting OTC availability of probiotics/prebiotics and pharmacists’ accessibility and frequent involvement in supplement counseling and safety screening.

# Methods:

## Sampling: Clearly state whether the sampling approach was random, convenience, or snowball.

**Response:** Thank you for highlighting this. We agree that the original Methods did not explicitly name the sampling approach. We have revised the manuscript to clearly state that recruitment used a non-probability sampling strategy (convenience sampling with snowball dissemination) through professional networks and online platforms (WhatsApp/social media groups).

**Change: we added the following to sampling method;**

Participants were recruited using a non-probability online approach (convenience sampling with snowball dissemination) through professional pharmacist networks, including WhatsApp groups and social media platforms. Because recruitment relied on online distribution and voluntary participation, selection bias and limited generalizability are possible.

## Given recruitment via social media and WhatsApp, potential selection bias should be acknowledged.

**Response:** Thank you. We agree that our original Methods did not explicitly name the sampling strategy. We have revised the manuscript to clearly state that recruitment used a non-probability online approach (convenience with snowball dissemination) via professional networks (WhatsApp/social media), and we explicitly acknowledge the risk of selection bias and limited generalizability. We also aligned reporting with STROBE guidance for cross-sectional studies by clarifying participant eligibility, recruitment channels, and bias consideration

**Change:** add a limitation point,

In addition, the survey was distributed online via email, WhatsApp, text messages, and pharmacist social media groups with snowball forwarding; therefore, the sample may over-represent pharmacists who are more digitally connected or more interested in probiotics/prebiotics, introducing potential selection bias and limiting generalizability.

## Questionnaire validation:The validation process is inadequately described. Stating that items were “in line with previous studies” does not constitute validation. Although a Content Validity Index (CVI) of 0.82 is reported, essential methodological details are missing, including:

### How the expert panel was selected

The number and expertise of panel members

The criteria used for the 4-point relevance scale

Whether pilot testing assessed reliability (e.g., internal consistency or test–retest reliability)

This represents a significant methodological gap.

# Results:

## Much of Table 1 presents non-significant demographic comparisons (e.g., gender, age, residency by knowledge level). These could be moved to supplementary material to improve focus on meaningful findings.

**Response:** Thank you for this observation. We agree that several comparisons in Table 1 are non-significant and may distract from the main findings. We have reformatted the Results and Table 1 to emphasize statistically relevant and clinically meaningful variables, and we have moved the non-significant descriptive comparisons to the Supplementary Material to improve clarity and focus.

## Limited analytical depth:The analysis stops at univariate associations. Multivariate approaches (e.g., logistic regression) are needed to identify independent predictors of adequate knowledge while controlling for potential confounders (e.g., education, years of experience).

**Response:** We agree. To strengthen the analytical approach and account for potential confounding, we have added a multivariable regression analysis to identify independent predictors of knowledge. The Statistical Analysis and Results sections were updated accordingly, and the adjusted findings are now reported in the revised regression table (with coefficients and 95% CIs), along with model-fit statistics.

Knowledge versus application:

The knowledge score heavily emphasizes strain identification (e.g., L. rhamnosus). Factual recall of strain names does not necessarily reflect clinically relevant knowledge regarding indications, benefits, or appropriate use. The analysis does not distinguish between factual knowledge and applied clinical understanding, which is more relevant to practice.

# Discussion

## Strengthen the rationale for why improving pharmacists’ knowledge is a public health priority in Palestine, linking it to patient safety, rational supplement use, and antimicrobial stewardship.

**Response:** We agree and have strengthened the Discussion to explicitly frame pharmacists’ probiotic/prebiotic counseling as a public health priority in Palestine, linking knowledge gaps to patient safety, rational supplement use, and counseling aligned with antimicrobial stewardship objectives.

**Change made:** Added a new rationale paragraph in the opening Discussion;

“This matters in Palestine because pharmacists are often the first point of contact for OTC supplement advice; strengthening pharmacists’ evidence-based knowledge supports patient safety, rational supplement use, and more appropriate counseling alongside antimicrobial stewardship goals (e.g., avoiding inappropriate recommendations in vulnerable groups and encouraging evidence-aligned use when indicated).”

## Expand interpretation of findings, particularly the reliance on internet and peer sources versus peer-reviewed literature, and relate this to continuing education needs.

**Response:** We agree and expanded interpretation of information sources to emphasize implications for evidence literacy, risk of misinformation, and the need for structured continuing professional development and curated evidence resources.

**Change made**: Expanded the “information sources” paragraph

“We expanded the interpretation of this finding because it has direct implications for continuing professional development: dependence on non-peer-reviewed sources may increase variability in counseling quality and increase the risk of misinformation”

## Contextualize limitations more fully, especially social desirability bias given high willingness to recommend probiotics.

**Response:** We agree and revised the limitations to explicitly note that self-report may introduce recall and social desirability bias, particularly given the high willingness to recommend probiotics.

**Change made:** Added a specific sentence on social desirability bias under limitations.

“Given the high reported willingness to recommend probiotics, social desirability bias may have inflated favorable attitude responses”

“Because recruitment used a non-probability approach, some characteristics were unevenly distributed (e.g., a higher proportion of younger participants and community pharmacists), which may limit representativeness across all pharmacist subgroups.”
