# Supplementary material for: Pharmacists Knowledge, Attitudes, and Practices Regarding Probiotics and Prebiotics: A Cross-Sectional Study from Palestine
Source: PLoS One. 2026 Jun 18;21(6):e0350648. doi: 10.1371/journal.pone.0350648 (PMC13278477; doi:10.1371/journal.pone.0350648)
Supplement: Attachment — Submitted filename: Reviewer report responses.docx [file pone.0350648.s009.docx]

# Date: 7 Apr. 26

# Re: Response to reviewers’ comments on the manuscript:

“Pharmacists Knowledge, Attitudes, and Practices Regarding Probiotics and Prebiotics: A Cross-Sectional Study from Palestine”.

The authors would like to thank the Editor for considering the manuscript for publication in Plos One. The authors would also like to thank the Reviewers for their time and constructive comments and suggestions to improve the quality of the manuscript. The following responses have been prepared to address the comments in a point-by-point fashion.

# Reviewer #1:

Thanks to the authors for answering my questions and comments in detail. I also have some minor comments and hope that they are helpful to the authors.

1. The reported multivariable linear regression model shows a very poor fit (R² = 0.034; Adjusted R² = −0.002; F(12,319) = 0.936, p = 0.511), indicating that the included predictors explain very little of the variance in knowledge scores. Furthermore, several categorical variables (e.g., education level, province, profile, working setting, and geographic location) were entered as numerically coded single-term predictors rather than as indicator (dummy) variables. As a result, the regression coefficients reflect the expected change in knowledge score per one-unit increase in the coding scale and cannot be interpreted as comparisons between specific categories and a reference group, which may be misleading, especially for nominal variables without inherent ordering. The analysis was conducted using complete-case (listwise deletion, n = 332), which may reduce statistical power and introduce bias if missing data are not completely at random.

The authors are strongly encouraged to recode categorical variables into dummy indicators, clearly specify reference groups, and reconsider the modeling approach, as the current presentation does not allow meaningful interpretation of the effects of categorical predictors.

**Response:** We thank the reviewer for this comment. We agree that the initial model, which treated categorical variables as numerically coded predictors, limited interpretability.

Accordingly, we re-conducted the multivariable regression analysis. All categorical variables were entered as factors and dummy-coded with clearly defined reference categories, allowing coefficients to be interpreted as comparisons between categories.

The revised model demonstrated modest explanatory power and was statistically significant overall (R² = 0.10; adjusted R² = −0.04; F(22, 324) = 1.63; p = 0.04). Several variables were significantly associated with knowledge score, including age, years of experience, working hours, and prescription volume.

**Changes:** We have updated the Abstract, Methods, Results, Table 5, and Discussion accordingly, and clarified the use of complete-case analysis as a limitation.

## 2. The authors need to ensure consistency across all tables regarding the number of decimal places reported for percentages. A uniform format (e.g., one or two decimal places) should be applied throughout the manuscript to improve clarity and readability.

**Response:** We thank the reviewer for this suggestion. We have revised the manuscript to improve consistency in the presentation of percentages across tables and text.

## 3. There are still some grammar mistakes throughout the manuscript. A thorough language revision by a professional English editor is strongly recommended. For example:

“Pharmacists were excluded if they are retired pharmacists...”

“The fourth section assess attitudes and practices towards...”

**Response:** We thank the reviewer for this comment. The manuscript has been carefully revised to improve grammar, clarity, and readability. Sentence structure, verb tense, and phrasing inconsistencies have been corrected throughout the manuscript.

# Reviewer #4:

Thank you for acknowledge my comments.

## Please state explicitly that your study followed guideline of STROBE in your study design paragraph.

**Response:** We thank the reviewer for this comment. We have now explicitly stated that the study was designed and reported in accordance with the STROBE guidelines for cross-sectional studies.
