# Supplementary material for: Pharmacists Knowledge, Attitudes, and Practices Regarding Probiotics and Prebiotics: A Cross-Sectional Study from Palestine
Source: PLoS One. 2026 Jun 18;21(6):e0350648. doi: 10.1371/journal.pone.0350648 (PMC13278477; doi:10.1371/journal.pone.0350648)
Supplement: Attachment — Submitted filename: Response report 18042026.docx [file pone.0350648.s010.docx]

# Date: 18 Apr. 26

# Re: Response to reviewers’ comments on the manuscript:

“Pharmacists Knowledge, Attitudes, and Practices Regarding Probiotics and Prebiotics: A Cross-Sectional Study from Palestine”.

The authors would like to thank the Editor for considering the manuscript for publication in Plos One. The authors would also like to thank the Reviewers for their time and constructive comments and suggestions to improve the quality of the manuscript. The following responses have been prepared to address the comments in a point-by-point fashion.

Reviewer Comment:

Reviewer #1: The authors conducted a multivariable linear regression with a large number of covariates; however, several concerns regarding model performance and interpretability remain.

First, the model's reported explanatory power is very limited (R² = 0.10), and, more importantly, the adjusted R² is negative (−0.04). This indicates that the fitted model does not improve upon a null model with no predictors and suggests potential overfitting due to the inclusion of a large number of covariates relative to the sample size. Including 22 predictors may have introduced substantial noise, thereby reducing the model’s overall reliability.

Second, although the overall F-test was statistically significant (p = 0.04), this result should be interpreted with caution. A statistically significant F-test in the presence of a negative adjusted R² may reflect marginal effects driven by a small subset of variables rather than a well-performing model. Therefore, the model's practical significance and robustness are questionable.

Third, the authors report several statistically significant predictors; however, given the low explanatory power and potential overfitting, these associations may not be stable. It would be helpful to assess the robustness of these findings using alternative approaches, such as variable selection techniques or model reduction strategies.

To improve the analysis, the authors are encouraged to:

1. Consider reducing the number of covariates based on theoretical justification or data-driven approaches.

2. Evaluate model stability (e.g., through sensitivity analyses or penalized regression methods).

3. Report additional diagnostics (e.g., multicollinearity, residual analysis) to support model validity.

**Response:**

We thank the reviewer for this detailed and insightful critique. We fully acknowledge the concerns raised regarding model overfitting, interpretability, and validity. In response, we have substantially revised the multivariable analysis to address these issues in a rigorous and systematic manner.

First, to address overfitting and improve interpretability, we replaced the original full model (22 covariates) with a reduced model including only variables selected based on strong theoretical relevance and empirical support from prior literature. The revised model includes probiotic/prebiotic education, age, gender, prescription volume, and practice setting. This approach aligns with recommended practices for parsimonious model specification and avoids unnecessary inclusion of weak or redundant predictors.

Second, the revised model demonstrated improved performance, with a positive adjusted R² (adjusted R² = 0.04) compared to the previously negative value (−0.04), indicating that the model now provides explanatory value beyond a null model. Although the overall explanatory power remains modest (R² = 0.06), this is consistent with the exploratory nature of KAP studies, where knowledge outcomes are influenced by multiple unmeasured behavioral and contextual factors.

Third, we conducted additional diagnostic assessments to ensure model validity. Multicollinearity was evaluated using variance inflation factors (VIF), with all values approximately equal to 1, indicating no evidence of collinearity among predictors. Residual diagnostics were also examined and did not suggest violations of model assumptions.

Fourth, to assess robustness, we performed sensitivity analyses by applying robust standard errors (HC1) to account for potential heteroscedasticity. The results remained consistent with the primary model, particularly with respect to the association between probiotic education and knowledge score, supporting the stability of the results.

Finally, we have revised the interpretation throughout the manuscript to reflect the limited explanatory power of the model and to avoid overstatement of findings. The results are now explicitly presented as exploratory associations rather than definitive predictors, consistent with the cross-sectional design and the descriptive purpose of the study.
