# Supplementary material for: Pharmacists Knowledge, Attitudes, and Practices Regarding Probiotics and Prebiotics: A Cross-Sectional Study from Palestine
Source: PLoS One. 2026 Jun 18;21(6):e0350648. doi: 10.1371/journal.pone.0350648 (PMC13278477; doi:10.1371/journal.pone.0350648)
Supplement: Peer review history

# Peer review: Decision Letter 0

**DOI:** 10.1371/journal.pone.0350648.r001

**Authors:** Awatif Al-Judaibi

11 Jan 2026

PONE-D-25-56829

Pharmacists Knowledge, Attitudes, and Practices Regarding Probiotics and Prebiotics: A Cross-Sectional Study from Palestine

PLOS One

Dear Dr.. Daraghmeh,

Thank you for submitting your manuscript to PLOS ONE. After careful consideration, we feel that it has merit but does not fully meet PLOS ONE’s publication criteria as it currently stands. Therefore, we invite you to submit a revised version of the manuscript that addresses the points raised during the review process.

Please submit your revised manuscript by Feb 25 2026 11:59PM. If you will need more time than this to complete your revisions, please reply to this message or contact the journal office at plosone@plos.org. When you're ready to submit your revision, log on to https://www.editorialmanager.com/pone/ and select the 'Submissions Needing Revision' folder to locate your manuscript file.

Please include the following items when submitting your revised manuscript:

- A letter that responds to each point raised by the academic editor and reviewer(s). You should upload this letter as a separate file labeled 'Response to Reviewers'.
- A marked-up copy of your manuscript that highlights changes made to the original version. You should upload this as a separate file labeled 'Revised Manuscript with Track Changes'.
- An unmarked version of your revised paper without tracked changes. You should upload this as a separate file labeled 'Manuscript'.

If you would like to make changes to your financial disclosure, please include your updated statement in your cover letter. Guidelines for resubmitting your figure files are available below the reviewer comments at the end of this letter.

If applicable, we recommend that you deposit your laboratory protocols in protocols.io to enhance the reproducibility of your results. Protocols.io assigns your protocol its own identifier (DOI) so that it can be cited independently in the future. For instructions see: https://journals.plos.org/plosone/s/submission-guidelines#loc-laboratory-protocols. Additionally, PLOS ONE offers an option for publishing peer-reviewed Lab Protocol articles, which describe protocols hosted on protocols.io. Read more information on sharing protocols at https://plos.org/protocols?utm_medium=editorial-email&utm_source=authorletters&utm_campaign=protocols.

We look forward to receiving your revised manuscript.

Kind regards,

Awatif Abid Al-Judaibi, PhD

Academic Editor

PLOS One

Journal Requirements:

When submitting your revision, we need you to address these additional requirements.

1. Please ensure that your manuscript meets PLOS ONE's style requirements, including those for file naming. The PLOS ONE style templates can be found at

https://journals.plos.org/plosone/s/file?id=wjVg/PLOSOne_formatting_sample_main_body.pdf and

https://journals.plos.org/plosone/s/file?id=ba62/PLOSOne_formatting_sample_title_authors_affiliations.pdf

2. Please provide additional details regarding participant consent. In the ethics statement in the Methods and online submission information, please ensure that you have specified (1) whether consent was informed and (2) what type you obtained (for instance, written or verbal, and if verbal, how it was documented and witnessed). If your study included minors, state whether you obtained consent from parents or guardians. If the need for consent was waived by the ethics committee, please include this information.

If you are reporting a retrospective study of medical records or archived samples, please ensure that you have discussed whether all data were fully anonymized before you accessed them and/or whether the IRB or ethics committee waived the requirement for informed consent. If patients provided informed written consent to have data from their medical records used in research, please include this information

3. We note that your Data Availability Statement is currently as follows: “All relevant data are within the manuscript and its Supporting Information files.”

Please confirm at this time whether or not your submission contains all raw data required to replicate the results of your study. Authors must share the “minimal data set” for their submission. PLOS defines the minimal data set to consist of the data required to replicate all study findings reported in the article, as well as related metadata and methods (https://journals.plos.org/plosone/s/data-availability#loc-minimal-data-set-definition).

For example, authors should submit the following data:

- The values behind the means, standard deviations and other measures reported;

- The values used to build graphs;

- The points extracted from images for analysis.

Authors do not need to submit their entire data set if only a portion of the data was used in the reported study.

If your submission does not contain these data, please either upload them as Supporting Information files or deposit them to a stable, public repository and provide us with the relevant URLs, DOIs, or accession numbers. For a list of recommended repositories, please see https://journals.plos.org/plosone/s/recommended-repositories.

If there are ethical or legal restrictions on sharing a de-identified data set, please explain them in detail (e.g., data contain potentially sensitive information, data are owned by a third-party organization, etc.) and who has imposed them (e.g., an ethics committee). Please also provide contact information for a data access committee, ethics committee, or other institutional body to which data requests may be sent. If data are owned by a third party, please indicate how others may request data access.

4. If the reviewer comments include a recommendation to cite specific previously published works, please review and evaluate these publications to determine whether they are relevant and should be cited. There is no requirement to cite these works unless the editor has indicated otherwise.

[Note: HTML markup is below. Please do not edit.]

Reviewers' comments:

Reviewer's Responses to Questions

**Comments to the Author**

1. Is the manuscript technically sound, and do the data support the conclusions?

The manuscript must describe a technically sound piece of scientific research with data that supports the conclusions. Experiments must have been conducted rigorously, with appropriate controls, replication, and sample sizes. The conclusions must be drawn appropriately based on the data presented.

Reviewer #1: Partly

Reviewer #2: No

**********

2. Has the statistical analysis been performed appropriately and rigorously?

Reviewer #1: No

Reviewer #2: No

**********

3. Have the authors made all data underlying the findings in their manuscript fully available?

The PLOS Data policy requires authors to make all data underlying the findings described in their manuscript fully available without restriction, with rare exception (please refer to the Data Availability Statement in the manuscript PDF file). The data should be provided as part of the manuscript or its supporting information, or deposited to a public repository. For example, in addition to summary statistics, the data points behind means, medians and variance measures should be available. If there are restrictions on publicly sharing data—e.g. participant privacy or use of data from a third party—those must be specified.

Reviewer #1: No

Reviewer #2: Yes

**********

4. Is the manuscript presented in an intelligible fashion and written in standard English?

PLOS ONE does not copyedit accepted manuscripts, so the language in submitted articles must be clear, correct, and unambiguous. Any typographical or grammatical errors should be corrected at revision, so please note any specific errors here.

Reviewer #1: No

Reviewer #2: Yes

**********

5. Review Comments to the Author

Please use the space provided to explain your answers to the questions above. You may also include additional comments for the author, including concerns about dual publication, research ethics, or publication ethics. (Please upload your review as an attachment if it exceeds 20,000 characters)

Reviewer #1: Thank you for inviting me to peer-review this interesting manuscript. I have some comments and hope that they are helpful to the authors.

Abstract

1. “Knowledge was assessed using a scoring system, participants were categorized as having poor (≤50), fair (50–70%), or good (≥70%) knowledge.”

Is 50% considered poor or fair? Is 70% considered fair or good?

Introduction

2. “Typically found in foods like chicory root, garlic, and onions, which promote the growth of beneficial gut bacteria [7].”

This is not a complete sentence.

Methods

3. “After conducting a comprehensive literature review, a self-administered questionnaire was created (Supplementary file 1).”

It is unclear where the authors’ Supplementary file is located.

Results

4. In Table 1, please pay attention to the groups of each variable. For example, for age, the authors have 5 groups, but age 50 is missing. For years of experience, the authors have 2 groups: 1–5 and 5–10; where should those with exactly 5 years of experience be placed? The variable "Employees at site" also misses the value 5.

5. “Associations of knowledge score and sociodemographic variables and”

And what?

6. “Table 4 presents the adjusted chi-square analysis of knowledge levels among pharmacists who reported using probiotics.”

It is unclear which Table 4 is being referred to.

Discussion

7. I see the authors aimed to evaluate the KAP of pharmacists about probiotics and prebiotics, but the practice section is not emphasized. The authors do not discuss this part, nor mention practice in the Abstract or Conclusions.

8. “A key strength of this study is its large and diverse sample of pharmacists from various regions, practice settings, and educational backgrounds allowing for a representative view in Palestinian context.”

Please review this sentence and add the limitations of convenience sampling. Since the authors collected samples online, many variables are unevenly distributed among groups. For example, age is concentrated in younger groups, mostly community pharmacists, and work experience is generally low.

9. “All relevant data are within the manuscript and its Supporting Information files.”

“The datasets used and/or analyzed during the current study are available from the corresponding author on reasonable request.”

Please double-check PLOS ONE’s policy on sharing raw data.

10. There are many spelling errors. Please check the manuscript.

Reviewer #2: The manuscript addresses a timely and relevant topic, and the overall cross-sectional design is appropriate for the stated aims. The data generally support the conclusion that pharmacists demonstrate moderate knowledge and positive attitudes toward probiotics and prebiotics. However, several methodological and conceptual issues limit the technical rigor of the study and weaken the strength of the conclusions.

Major concerns:

Introduction:

The discussion of the therapeutic potential of probiotics (e.g., via the gut–brain axis) is overly brief and lacks sufficient critical context. This section would benefit from expansion using key references and a clearer rationale for why pharmacists—rather than, or in addition to, dietitians and gastroenterologists—require this knowledge. Explicitly acknowledging the primary role of dietitians in nutritional counseling would strengthen the justification for why pharmacists’ knowledge may be comparatively limited yet still clinically important given their accessibility and frequent role in over-the-counter (OTC) recommendations.

Methods:

Sampling: Clearly state whether the sampling approach was random, convenience, or snowball. Given recruitment via social media and WhatsApp, potential selection bias should be acknowledged.

Questionnaire validation:

The validation process is inadequately described. Stating that items were “in line with previous studies” does not constitute validation. Although a Content Validity Index (CVI) of 0.82 is reported, essential methodological details are missing, including:

How the expert panel was selected

The number and expertise of panel members

The criteria used for the 4-point relevance scale

Whether pilot testing assessed reliability (e.g., internal consistency or test–retest reliability)

This represents a significant methodological gap.

Results:

Much of Table 1 presents non-significant demographic comparisons (e.g., gender, age, residency by knowledge level). These could be moved to supplementary material to improve focus on meaningful findings.

Limited analytical depth:

The analysis stops at univariate associations. Multivariate approaches (e.g., logistic regression) are needed to identify independent predictors of adequate knowledge while controlling for potential confounders (e.g., education, years of experience).

Knowledge versus application:

The knowledge score heavily emphasizes strain identification (e.g., L. rhamnosus). Factual recall of strain names does not necessarily reflect clinically relevant knowledge regarding indications, benefits, or appropriate use. The analysis does not distinguish between factual knowledge and applied clinical understanding, which is more relevant to practice.

Discussion

Strengthen the rationale for why improving pharmacists’ knowledge is a public health priority in Palestine, linking it to patient safety, rational supplement use, and antimicrobial stewardship.

Expand interpretation of findings, particularly the reliance on internet and peer sources versus peer-reviewed literature, and relate this to continuing education needs.

Contextualize limitations more fully, especially social desirability bias given high willingness to recommend probiotics.

**********

6. PLOS authors have the option to publish the peer review history of their article (what does this mean?). If published, this will include your full peer review and any attached files.

If you choose “no”, your identity will remain anonymous but your review may still be made public.

**Do you want your identity to be public for this peer review?**  For information about this choice, including consent withdrawal, please see our Privacy Policy.

Reviewer #1: No

Reviewer #2: No

**********

[NOTE: If reviewer comments were submitted as an attachment file, they will be attached to this email and accessible via the submission site. Please log into your account, locate the manuscript record, and check for the action link "View Attachments". If this link does not appear, there are no attachment files.]

To ensure your figures meet our technical requirements, please review our figure guidelines: https://journals.plos.org/plosone/s/figures

You may also use PLOS’s free figure tool, NAAS, to help you prepare publication quality figures: https://journals.plos.org/plosone/s/figures#loc-tools-for-figure-preparation.

NAAS will assess whether your figures meet our technical requirements by comparing each figure against our figure specifications.

# Author comment: Author response to Decision Letter 1

**DOI:** 10.1371/journal.pone.0350648.r002

8 Feb 2026

Date: 23rd January 2026

Re: Response to reviewers’ comments on the manuscript:

“Pharmacists Knowledge, Attitudes, and Practices Regarding Probiotics and Prebiotics: A Cross-Sectional Study from Palestine”.

The authors would like to thank the Editor for considering the manuscript for publication in PLOS ONE. The authors would also like to thank the Reviewers for their time and constructive comments and suggestions to improve the quality of the manuscript. The following responses have been prepared to address the comments in a point-by-point fashion.

Reviewers' comments:

Reviewer #1:

Abstract

“Knowledge was assessed using a scoring system, participants were categorized as having poor (≤50), fair (50–70%), or good (≥70%) knowledge.” Is 50% considered poor or fair? Is 70% considered fair or good?

Response: Correct—this was ambiguous due to overlapping boundaries. In Methods we define the cutoffs explicitly (≤50%, 50–70%, ≥70%), but the Abstract wording caused overlap.

Change: Abstract revised to use non-overlapping thresholds:

“poor (≤50%; 0–17 points), moderate (>50% to <70%; 18–23 points), or high (≥70%; 24–34 points)”

Introduction

“Typically found in foods like chicory root, garlic, and onions, which promote respthe growth of beneficial gut bacteria [7].” This is not a complete sentence.

Response: Thank you for noting this. We agree the sentence was a fragment and also contained a typographical error (“respthe”). We revised the wording to form a complete sentence and corrected the typo while maintaining the intended meaning and citation.

Change: Revision made in manuscript:

“Prebiotics are non-digestible substrates that are selectively utilized by host microorganisms and may confer health benefits; they are naturally present in foods such as chicory root, garlic, and onions, which can promote the growth of beneficial gut bacteria [7]”.

Methods

“After conducting a comprehensive literature review, a self-administered questionnaire was created (Supplementary file 1).” sIt is unclear where the authors’ Supplementary file is located.

Response: We agree and have clarified this. We have uploaded the questionnaire as Supporting Information and revised the Methods text to explicitly indicate its location and label in the manuscript. The questionnaire is now provided as Supplementary file 1 and is referenced consistently throughout the manuscript.

Results

In Table 1, please pay attention to the groups of each variable. For example, for age, the authors have 5 groups, but age 50 is missing. For years of experience, the authors have 2 groups: 1–5 and 5–10; where should those with exactly 5 years of experience be placed? The variable "Employees at site" also misses the value

Response: Thank you for pointing this boundary-definition errors. We revised Table 1 to ensure all categories are mutually exclusive and collectively exhaustive, and we clarified category labels to explicitly include boundary values.

“Associations of knowledge score and sociodemographic variables and” And what?

Response: We agree and corrected the truncated subtitle.

Change: Revised the subtitle to: “Associations between knowledge score and sociodemographic and professional characteristics.”

Table 4 presents the adjusted chi-square analysis of knowledge levels among pharmacists who reported using probiotics.

This section has been modified, and the results are now presented in Table 5.

It is unclear which Table 4 is being referred to.

Response: We agree. This was due to an incorrect table numbering/cross-reference in the Results section. We have corrected the table numbering throughout the manuscript and updated the in-text citation to refer to the correct table. All table callouts and captions were verified to ensure consistency.

Discussion

I see the authors aimed to evaluate the KAP of pharmacists about probiotics and prebiotics, but the practice section is not emphasized. The authors do not discuss this part, nor mention practice in the Abstract or Conclusions.

Response: Thank you for this important observation. We agree that the initial version under-emphasized the practice component. We have revised the manuscript to explicitly include practice in the Abstract and Conclusion, and expand the Discussion to address pharmacists’ self-reported counseling/recommendation practices and barriers, linking these findings to practice-oriented educational needs.

“A key strength of this study is its large and diverse sample of pharmacists from various regions, practice settings, and educational backgrounds allowing for a representative view in Palestinian context.” Please review this sentence and add the limitations of convenience sampling. Since the authors collected samples online, many variables are unevenly distributed among groups. For example, age is concentrated in younger groups, mostly community pharmacists, and work experience is generally low.

Response: Thank you for this comment. We agree that the original wording overstated representativeness. We revised the strength statement and explicitly added the limitations related to online non-probability recruitment (convenience sampling with snowball dissemination), including potential selection bias, uneven distribution of participant characteristics (predominance of younger pharmacists and community pharmacy respondents), and generally lower work experience, which may limit generalizability.

“All relevant data are within the manuscript and its Supporting Information files.” “The datasets used and/or analyzed during the current study are available from the corresponding author on reasonable request.” Please double-check PLOS ONE’s policy on sharing raw data.

Response: Thank you for raising this. We agree that the original statements did not adequately reflect PLOS ONE’s Data Availability requirements. PLOS ONE requires that the data underlying the findings be made available, and when public sharing is not possible due to ethical/legal restrictions, authors must state the restriction and provide a clear access route (e.g., through an ethics committee/data access contact), rather than only “author on request.”

Accordingly, we revised the Data Availability statement to clarify the ethical restriction and to specify an institutional access pathway via the Al-Quds University Research Ethics Committee.

Change: Data Availability: The data collected and analyzed during this study are not publicly accessible in accordance with the Rules Governing the Ethics of Scientific Research. However, researchers interested in obtaining this data may contact the IRB Coordinator at Al-Quds University via email at Research@admin.alquds.edu.

There are many spelling errors. Please check the manuscript.

Response: Thank you for pointing this out. We performed a comprehensive language edit of the entire manuscript to correct spelling, grammar, punctuation, and phrasing. We also checked consistency of terminology and removed duplicated or awkward wording to improve readability.

Reviewer #2:

The manuscript addresses a timely and relevant topic, and the overall cross-sectional design is appropriate for the stated aims. The data generally support the conclusion that pharmacists demonstrate moderate knowledge and positive attitudes toward probiotics and prebiotics. However, several methodological and conceptual issues limit the technical rigor of the study and weaken the strength of the conclusions.

Introduction:

The discussion of the therapeutic potential of probiotics (e.g., via the gut–brain axis) is overly brief and lacks sufficient critical context. This section would benefit from expansion using key references and a clearer rationale for why pharmacists—rather than, or in addition to, dietitians and gastroenterologists—require this knowledge. Explicitly acknowledging the primary role of dietitians in nutritional counseling would strengthen the justification for why pharmacists’ knowledge may be comparatively limited yet still clinically important given their accessibility and frequent role in over-the-counter (OTC) recommendations.

Response: Thank you for this valuable suggestion. We agree that the Introduction required stronger scientific context and a clearer rationale for pharmacists’ relevance. We expanded the Introduction to (1) define probiotics and prebiotics using widely accepted consensus definitions, (2) briefly contextualize the microbiota–gut–brain axis and emphasize that evidence is evolving and often strain/indication-specific, (3) acknowledge that dietitians are primary providers of nutrition counseling, and (4) justify pharmacists’ importance because probiotics/prebiotics are frequently obtained over-the-counter and pharmacists are highly accessible healthcare professionals who counsel on supplements and self-care products.

Change: revision made to manuscript;

• Expanded the Introduction to include consensus definitions of probiotics/prebiotics/synbiotics and to add concise context on the microbiota–gut–brain axis with an evidence-cautious framing.

• Added explicit acknowledgement that dietitians are primary providers of nutrition counseling and that gastroenterologists often lead gastrointestinal clinical management.

• Added a clear rationale for pharmacists’ relevance by highlighting OTC availability of probiotics/prebiotics and pharmacists’ accessibility and frequent involvement in supplement counseling and safety screening.

Methods:

Sampling: Clearly state whether the sampling approach was random, convenience, or snowball.

Response: Thank you for highlighting this. We agree that the original Methods did not explicitly name the sampling approach. We have revised the manuscript to clearly state that recruitment used a non-probability sampling strategy (convenience sampling with snowball dissemination) through professional networks and online platforms (WhatsApp/social media groups).

Change: we added the following to sampling method;

Participants were recruited using a non-probability online approach (convenience sampling with snowball dissemination) through professional pharmacist networks, including WhatsApp groups and social media platforms. Because recruitment relied on online distribution and voluntary participation, selection bias and limited generalizability are possible.

Given recruitment via social media and WhatsApp, potential selection bias should be acknowledged.

Response: Thank you. We agree that our original Methods did not explicitly name the sampling strategy. We have revised the manuscript to clearly state that recruitment used a non-probability online approach (convenience with snowball dissemination) via professional networks (WhatsApp/social media), and we explicitly acknowledge the risk of selection bias and limited generalizability. We also aligned reporting with STROBE guidance for cross-sectional studies by clarifying participant eligibility, recruitment channels, and bias consideration

Change: add a limitation point,

In addition, the survey was distributed online via email, WhatsApp, text messages, and pharmacist social media groups with snowball forwarding; therefore, the sample may over-represent pharmacists who are more digitally connected or more interested in probiotics/prebiotics, introducing potential selection bias and limiting generalizability.

Questionnaire validation:The validation process is inadequately described. Stating that items were “in line with previous studies” does not constitute validation. Although a Content Validity Index (CVI) of 0.82 is reported, essential methodological details are missing, including:

How the expert panel was selected

The number and expertise of panel members

The criteria used for the 4-point relevance scale

Whether pilot testing assessed reliability (e.g., internal consistency or test–retest reliability)

This represents a significant methodological gap.

Results:

Much of Table 1 presents non-significant demographic comparisons (e.g., gender, age, residency by knowledge level). These could be moved to supplementary material to improve focus on meaningful findings.

Response: Thank you for this observation. We agree that several comparisons in Table 1 are non-significant and may distract from the main findings. We have reformatted the Results and Table 1 to emphasize statistically relevant and clinically meaningful variables, and we have moved the non-significant descriptive comparisons to the Supplementary Material to improve clarity and focus.

Limited analytical depth:The analysis stops at univariate associations. Multivariate approaches (e.g., logistic regression) are needed to identify independent predictors of adequate knowledge while controlling for potential confounders (e.g., education, years of experience).

Response: We agree. To strengthen the analytical approach and account for potential confounding, we have added a multivariable regression analysis to identify independent predictors of knowledge. The Statistical Analysis and Results sections were updated accordingly, and the adjusted findings are now reported in the revised regression table (with coefficients and 95% CIs), along with model-fit statistics.

Knowledge versus application:

The knowledge score heavily emphasizes strain identification (e.g., L. rhamnosus). Factual recall of strain names does not necessarily reflect clinically relevant knowledge regarding indications, benefits, or appropriate use. The analysis does not distinguish between factual knowledge and applied clinical understanding, which is more relevant to practice.

Discussion

Strengthen the rationale for why improving pharmacists’ knowledge is a public health priority in Palestine, linking it to patient safety, rational supplement use, and antimicrobial stewardship.

Response: We agree and have strengthened the Discussion to explicitly frame pharmacists’ probiotic/prebiotic counseling as a public health priority in Palestine, linking knowledge gaps to patient safety, rational supplement use, and counseling aligned with antimicrobial stewardship objectives.

Change made: Added a new rationale paragraph in the opening Discussion;

“This matters in Palestine because pharmacists are often the first point of contact for OTC supplement advice; strengthening pharmacists’ evidence-based knowledge supports patient safety, rational supplement use, and more appropriate counseling alongside antimicrobial stewardship goals (e.g., avoiding inappropriate recommendations in vulnerable groups and encouraging evidence-aligned use when indicated).”

Expand interpretation of findings, particularly the reliance on internet and peer sources versus peer-reviewed literature, and relate this to continuing education needs.

Response: We agree and expanded interpretation of information sources to emphasize implications for evidence literacy, risk of misinformation, and the need for structured continuing professional development and curated evidence resources.

Change made: Expanded the “information sources” paragraph

“We expanded the interpretation of this finding because it has direct implications for continuing professional development: dependence on non-peer-reviewed sources may increase variability in counseling quality and increase the risk of misinformation”

Contextualize limitations more fully, especially social desirability bias given high willingness to recommend probiotics.

Response: We agree and revised the limitations to explicitly note that self-report may introduce recall and social desirability bias, particularly given the high willingness to recommend probiotics.

Change made: Added a specific sentence on social desirability bias under limitations.

“Given the high reported willingness to recommend probiotics, social desirability bias may have inflated favorable attitude responses”

“Because recruitment used a non-probability approach, some characteristics were unevenly distributed (e.g., a higher proportion of younger participants and community pharmacists), which may limit representativeness across all pharmacist subgroups.”

Attachment Submitted filename: Reviewer Report-DD.docx

# Peer review: Decision Letter 1

**DOI:** 10.1371/journal.pone.0350648.r003

**Authors:** Awatif Al-Judaibi, Awatif Al-Judaibi

27 Feb 2026

PONE-D-25-56829R1

Pharmacists Knowledge, Attitudes, and Practices Regarding Probiotics and Prebiotics: A Cross-Sectional Study from Palestine

PLOS One

Dear Dr. Daraghmeh,

Thank you for submitting your manuscript to PLOS ONE. After careful consideration, we feel that it has merit but does not fully meet PLOS ONE’s publication criteria as it currently stands. Therefore, we invite you to submit a revised version of the manuscript that addresses the points raised during the review process.

Please submit your revised manuscript by Apr 13 2026 11:59PM. If you will need more time than this to complete your revisions, please reply to this message or contact the journal office at plosone@plos.org. When you're ready to submit your revision, log on to https://www.editorialmanager.com/pone/ and select the 'Submissions Needing Revision' folder to locate your manuscript file.

Please include the following items when submitting your revised manuscript:

- A letter that responds to each point raised by the academic editor and reviewer(s). You should upload this letter as a separate file labeled 'Response to Reviewers'.
- A marked-up copy of your manuscript that highlights changes made to the original version. You should upload this as a separate file labeled 'Revised Manuscript with Track Changes'.
- An unmarked version of your revised paper without tracked changes. You should upload this as a separate file labeled 'Manuscript'.

-->If you would like to make changes to your financial disclosure, please include your updated statement in your cover letter. Guidelines for resubmitting your figure files are available below the reviewer comments at the end of this letter.

If applicable, we recommend that you deposit your laboratory protocols in protocols.io to enhance the reproducibility of your results. Protocols.io assigns your protocol its own identifier (DOI) so that it can be cited independently in the future. For instructions see: https://journals.plos.org/plosone/s/submission-guidelines#loc-laboratory-protocols. Additionally, PLOS ONE offers an option for publishing peer-reviewed Lab Protocol articles, which describe protocols hosted on protocols.io. Read more information on sharing protocols at https://plos.org/protocols?utm_medium=editorial-email&utm_source=authorletters&utm_campaign=protocols.

We look forward to receiving your revised manuscript.

Kind regards,

Awatif Abid Al-Judaibi, PhD

Academic Editor

PLOS One

**Journal Requirements:**

1. If the reviewer comments include a recommendation to cite specific previously published works, please review and evaluate these publications to determine whether they are relevant and should be cited. There is no requirement to cite these works unless the editor has indicated otherwise.

2. Please review your reference list to ensure that it is complete and correct. If you have cited papers that have been retracted, please include the rationale for doing so in the manuscript text, or remove these references and replace them with relevant current references. Any changes to the reference list should be mentioned in the rebuttal letter that accompanies your revised manuscript. If you need to cite a retracted article, indicate the article’s retracted status in the References list and also include a citation and full reference for the retraction notice.

[Note: HTML markup is below. Please do not edit.]

Reviewers' comments:

Reviewer's Responses to Questions

**Comments to the Author**

1. If the authors have adequately addressed your comments raised in a previous round of review and you feel that this manuscript is now acceptable for publication, you may indicate that here to bypass the “Comments to the Author” section, enter your conflict of interest statement in the “Confidential to Editor” section, and submit your "Accept" recommendation.

Reviewer #1: (No Response)

Reviewer #3: All comments have been addressed

Reviewer #4: (No Response)

**********

2. Is the manuscript technically sound, and do the data support the conclusions?

The manuscript must describe a technically sound piece of scientific research with data that supports the conclusions. Experiments must have been conducted rigorously, with appropriate controls, replication, and sample sizes. The conclusions must be drawn appropriately based on the data presented.

Reviewer #1: Partly

Reviewer #3: Yes

Reviewer #4: Yes

**********

3. Has the statistical analysis been performed appropriately and rigorously?

Reviewer #1: No

Reviewer #3: Yes

Reviewer #4: Yes

**********

4. Have the authors made all data underlying the findings in their manuscript fully available?

The PLOS Data policy requires authors to make all data underlying the findings described in their manuscript fully available without restriction, with rare exception (please refer to the Data Availability Statement in the manuscript PDF file). The data should be provided as part of the manuscript or its supporting information, or deposited to a public repository. For example, in addition to summary statistics, the data points behind means, medians and variance measures should be available. If there are restrictions on publicly sharing data—e.g. participant privacy or use of data from a third party—those must be specified.

Reviewer #1: Yes

Reviewer #3: Yes

Reviewer #4: Yes

**********

5. Is the manuscript presented in an intelligible fashion and written in standard English?

PLOS ONE does not copyedit accepted manuscripts, so the language in submitted articles must be clear, correct, and unambiguous. Any typographical or grammatical errors should be corrected at revision, so please note any specific errors here.

Reviewer #1: No

Reviewer #3: Yes

Reviewer #4: Yes

**********

6. Review Comments to the Author

Please use the space provided to explain your answers to the questions above. You may also include additional comments for the author, including concerns about dual publication, research ethics, or publication ethics. (Please upload your review as an attachment if it exceeds 20,000 characters)

Reviewer #1: Abstract

1. The study objectives are described inconsistently throughout the manuscript. For example:

“This study aimed to assess pharmacists’ KAP related to probiotics and prebiotics and to identify demographic and professional factors influencing knowledge.”

“Therefore, this study aimed to assess the knowledge, attitudes, and practices of pharmacists in Palestine regarding probiotics and prebiotics, and to identify factors that may influence these domains.”

“The statistical analysis employed included both descriptive and inferential statistics to provide a comprehensive understanding of the factors influencing pharmacists professionals' knowledge and attitudes toward probiotics and prebiotics.”

The authors should ensure that the study objective is clearly defined and consistently stated throughout the manuscript.

2. “The mean knowledge score was 21.3 ± 6.1 (range: 4-34). Most participants demonstrated moderate knowledge (57.8%), while 32.0% achieved high knowledge and 10.3% had poor knowledge. Attitudes were generally positive, with 85.5% believed in their health benefits and 81% were willing to recommend them. In multivariable analysis, years of experience was independently associated with knowledge score (p= 0.027). Practice findings indicated variable self-reported counseling and recommendation behaviors, with barriers related to evidence access and confidence in clinical application.”

In the Abstract, the results are not presented in a fully consistent KAP sequence. The findings should be reported systematically in the following order: knowledge, attitudes, and then practices. A similar structural issue is observed in the Discussion section, where the interpretation of results should also follow the same logical order for clarity and coherence.

Methods

3. “Because recruitment relied on online distribution and voluntary participation, selection bias and limited generalizability are possible.”

This reflects a study limitation and should therefore be moved to the Discussion section under the limitations subsection, rather than appearing in the Methods.

4. In the Statistical Analysis section, the authors do not describe the use of univariable and multivariable regression models, nor do they explain the criteria for variable selection in the multivariable model (e.g., p-value threshold, theoretical relevance, stepwise approach, etc.). These methodological details are essential for transparency and reproducibility and should be clearly reported.

Results

5. “The majority of participants were female (66.3%), aged between 20 and 29 (74.3%)”

“With 57% reported no formal training, 28.5% indicated they had received some education, and 17% were unsure of their knowledge.”

The authors should ensure that the numerical data presented in the text are fully consistent with those reported in the corresponding tables.

6. Several rows in Table 1 do not include percentages. Additionally, the number of decimal places is not consistent across the manuscript. The authors should standardize the reporting format throughout the paper.

7. In Table 1, for the variable “Pharmacy open hours/week,” the category structure appears incomplete. There seems to be a missing category between >120 hours and <7 days. The authors should review and clarify the categorization.

8. In Table 2, the response categories should be arranged in descending order of percentage to improve readability and facilitate interpretation.

9. In Table 3, the total number of responses in at least one row does not equal 400. The authors should verify and correct the calculations to ensure internal consistency.

10. Regarding Table 5:

The reported regression results indicate a very low explanatory power (R² = 0.034), and more importantly, a negative adjusted R² (Adjusted R² = −0.002). A negative adjusted R² suggests that, after accounting for the number of predictors in the model, the model performs worse than an intercept-only model. In other words, the included independent variables do not meaningfully improve the prediction of the dependent variable. This raises concerns regarding the adequacy of the model specification and the theoretical relevance of the selected predictors. The authors are encouraged to re-examine the model, justify the inclusion of the predictors, and discuss the implications of this extremely low explanatory power. At minimum, this limitation should be explicitly acknowledged in the manuscript.

In addition, the structure of Table 5 is unclear. For categorical variables, one category is typically selected as the reference group, and regression coefficients are presented for the remaining categories. However, in this table, each categorical variable appears to be represented by only one row. For example, in Table 1, education level is divided into four categories (Bachelor, PharmD, Master, and PhD), yet in Table 5, only a single row is shown for this variable. The authors should clarify how categorical variables were coded and analyzed in the regression model.

11. There are still numerous typographical errors and awkward expressions throughout the manuscript. A thorough language revision by a professional English editor is strongly recommended.

Reviewer #3: (No Response)

Reviewer #4: This is a good study regarding KAP, and helps to inform improvement of pharmacists practice regarding probiotics and prebiotics. Here are my comments:

1. Knowledge score of 50% fall in poor and fair & 70% in fair & knowledgeable as mention in abstract and statistical analysis - 1. Poor Knowledge: 50% or less (≤ 12 points); 2. Fair Knowledge: Scores between 50% and 70% (13-24 points); 3. Knowledgeable: Scores of 70% or higher (≥ 25 points). It should have clear cut off point between these scores.

2. As this is cross sectional study please consider include reporting findings accordance to STROBE in study design.

3. “Questionnaire was reviewed by a panel of specialists in the fields of pharmacy”- Please indicate how many panels were involved for this validity.

4. Was the pilot study conducted online to 20 respondents? Please state in procedure

5. Data analysis part- Is there any missing data and how authors handled missing data from respondents?

6. Results – Please include mean,SD of respondents age for better clarity of demographic respondents.

7. Please do not repeat the result values in discussion section. E.g (57.8%), 32.0%, 10.3%

**********

7. PLOS authors have the option to publish the peer review history of their article (what does this mean?). If published, this will include your full peer review and any attached files.

If you choose “no”, your identity will remain anonymous but your review may still be made public.

**Do you want your identity to be public for this peer review?**  For information about this choice, including consent withdrawal, please see our Privacy Policy.

Reviewer #1: No

Reviewer #3: No

Reviewer #4: No

**********

[NOTE: If reviewer comments were submitted as an attachment file, they will be attached to this email and accessible via the submission site. Please log into your account, locate the manuscript record, and check for the action link "View Attachments". If this link does not appear, there are no attachment files.]

To ensure your figures meet our technical requirements, please review our figure guidelines: https://journals.plos.org/plosone/s/figures

You may also use PLOS’s free figure tool, NAAS, to help you prepare publication quality figures: https://journals.plos.org/plosone/s/figures#loc-tools-for-figure-preparation.

NAAS will assess whether your figures meet our technical requirements by comparing each figure against our figure specifications.

# Author comment: Author response to Decision Letter 2

**DOI:** 10.1371/journal.pone.0350648.r004

2 Mar 2026

Date: 2 Mar. 26

Re: Response to reviewers’ comments on the manuscript:

“Pharmacists Knowledge, Attitudes, and Practices Regarding Probiotics and Prebiotics: A Cross-Sectional Study from Palestine”.

The authors would like to thank the Editor for considering the manuscript for publication in PLOS ONE. The authors would also like to thank the Reviewers for their time and constructive comments and suggestions to improve the quality of the manuscript. The following responses have been prepared to address the comments in a point-by-point fashion.

Reviewers' comments:

Reviewer #1:

Abstract

1. The study objectives are described inconsistently throughout the manuscript. For example:

“This study aimed to assess pharmacists’ KAP related to probiotics and prebiotics and to identify demographic and professional factors influencing knowledge.”

“Therefore, this study aimed to assess the knowledge, attitudes, and practices of pharmacists in Palestine regarding probiotics and prebiotics, and to identify factors that may influence these domains.”

“The statistical analysis employed included both descriptive and inferential statistics to provide a comprehensive understanding of the factors influencing pharmacists professionals' knowledge and attitudes toward probiotics and prebiotics.”

The authors should ensure that the study objective is clearly defined and consistently stated throughout the manuscript.

Response: We thank the reviewer for this constructive comment.

Revised standardized objective (now used consistently):

“To assess pharmacists’ knowledge, attitudes, and practices (KAP) regarding probiotics and prebiotics in Palestine, and to identify demographic and professional factors associated with pharmacists’ knowledge score.”

Where revised:

Abstract: Aim sentence revised to match the standardized objective exactly.

Introduction (last paragraph): Objective sentence revised to match verbatim.

Methods/Statistical Analysis: Removed wording implying analysis of “factors influencing attitudes” and clarified that multivariable analysis examined factors associated with knowledge score (with attitudes and practices summarized descriptively).

2. “The mean knowledge score was 21.3 ± 6.1 (range: 4-34). Most participants demonstrated moderate knowledge (57.8%), while 32.0% achieved high knowledge and 10.3% had poor knowledge. Attitudes were generally positive, with 85.5% believed in their health benefits and 81% were willing to recommend them. In multivariable analysis, years of experience was independently associated with knowledge score (p= 0.027). Practice findings indicated variable self-reported counseling and recommendation behaviors, with barriers related to evidence access and confidence in clinical application.”

In the Abstract, the results are not presented in a fully consistent KAP sequence. The findings should be reported systematically in the following order: knowledge, attitudes, and then practices. A similar structural issue is observed in the Discussion section, where the interpretation of results should also follow the same logical order for clarity and coherence.

Response: Thank you for this helpful suggestion. For clarity and coherence, we have restructured both sections (Results- Abstract and discussion) to present and interpret findings systematically in the KAP order.

Methods

3. “Because recruitent relied on online distribution and voluntary participation, selection bias and limited generalizability are possible.”

This reflects a study limitation and should therefore be moved to the Discussion section under the limitations subsection, rather than appearing in the Methods.

Response: Thank you for this observation. We agree that the statement regarding selection bias and generalizability is a study limitation and should not appear in the Methods section.

Change made: We deleted this sentence from the Methods and moved it to the Discussion under the Limitations subsection.

4. In the Statistical Analysis section, the authors do not describe the use of univariable and multivariable regression models, nor do they explain the criteria for variable selection in the multivariable model (e.g., p-value threshold, theoretical relevance, stepwise approach, etc.). These methodological details are essential for transparency and reproducibility and should be clearly reported.

Response: Thank you for this comment. We revised the Statistical Analysis section to explicitly report univariable and multivariable regression models

Results

5. “The majority of participants were female (66.3%), aged between 20 and 29 (74.3%)”

“With 57% reported no formal training, 28.5% indicated they had received some education, and 17% were unsure of their knowledge.”

The authors should ensure that the numerical data presented in the text are fully consistent with those reported in the corresponding tables.

Response: Thank you for pointing this out. We agree that all numerical values reported in the narrative must match the corresponding tables exactly to avoid confusion and to ensure internal consistency.

Change made: We re-checked all demographic and training-related figures in the Results text against the values in Table 1 (and other relevant tables) and corrected any discrepancies. Specifically, we ensured that the reported proportions for sex, age group (20–29 years), and formal training/education regarding probiotics and prebiotics are now identical in both the text and the tables. Where needed, we also standardized rounding and decimal places to match the tables.

6. Several rows in Table 1 do not include percentages. Additionally, the number of decimal places is not consistent across the manuscript. The authors should standardize the reporting format throughout the paper.

Response: Agreed. Presentation consistency matters.

Change made: Added missing percentages to Table 1 rows where counts were provided without percentages.

Standardized percent formatting across tables (consistent decimal places throughout).

7. In Table 1, for the variable “Pharmacy open hours/week,” the category structure appears incomplete. There seems to be a missing category between >120 hours and <7 days. The authors should review and clarify the categorization.

Response: We understand the concern. The intention was to represent three operational patterns (<80, 80–120, and 24/7). The third category corresponds to >120 hours/week.

Change made: We revised the label to an unambiguous form:

“24/7 (7 days × 24 h/day; >120 h/week)”

This makes the implied weekly-hours meaning explicit and removes the appearance of a missing interval.

8. In Table 2, the response categories should be arranged in descending order of percentage to improve readability and facilitate interpretation.

Response: Thank you for this comment. Agreed.

Change made: Table 2 response categories were reordered from highest to lowest percentage to improve interpretability.

9. In Table 3, the total number of responses in at least one row does not equal 400. The authors should verify and correct the calculations to ensure internal consistency.

Response: Thank you for noting this issue. We agree that the original Table 3 contained an internal inconsistency in which the total number of responses for some knowledge items did not equal the study sample size (N = 400). This occurred due to an error in how the table was compiled, which led to incorrect row totals.

Change made: We have corrected Table 3 by re-generating the frequency table directly from the final dataset using the full sample as the denominator (N = 400) and ensuring that each knowledge item has mutually exclusive response categories (Correct / Incorrect / Not sure) that sum to 400. Percentages were recalculated accordingly as n/400 × 100. The revised table now shows consistent totals (400) for every row and internally consistent percentages.

10. Regarding Table 5:

The reported regression results indicate a very low explanatory power (R² = 0.034), and more importantly, a negative adjusted R² (Adjusted R² = −0.002). A negative adjusted R² suggests that, after accounting for the number of predictors in the model, the model performs worse than an intercept-only model. In other words, the included independent variables do not meaningfully improve the prediction of the dependent variable. This raises concerns regarding the adequacy of the model specification and the theoretical relevance of the selected predictors. The authors are encouraged to re-examine the model, justify the inclusion of the predictors, and discuss the implications of this extremely low explanatory power. At minimum, this limitation should be explicitly acknowledged in the manuscript.

In addition, the structure of Table 5 is unclear. For categorical variables, one category is typically selected as the reference group, and regression coefficients are presented for the remaining categories. However, in this table, each categorical variable appears to be represented by only one row. For example, in Table 1, education level is divided into four categories (Bachelor, PharmD, Master, and PhD), yet in Table 5, only a single row is shown for this variable. The authors should clarify how categorical variables were coded and analyzed in the regression model.

Response: Thank you for this important comment. We agree that the model has very limited explanatory power (R² = 0.034; adjusted R² = −0.002), and we have explicitly acknowledged this limitation in the manuscript. We also agree that the original Table 5 presentation did not clearly explain how categorical predictors were handled, which could lead to misinterpretation.

Clarification (categorical coding): In the regression model, several categorical variables (e.g., education level, province, profile, working setting, and geographic location) were entered into SPSS using their numeric coding as single predictors rather than being expanded into indicator (dummy) variables with an explicit reference category. Therefore, the coefficients shown for these variables represent the expected change in knowledge score per one-unit increase in the coding scale, and do not represent category-versus-reference comparisons.

Change made (Table 5 and Methods): To ensure transparency and reproducibility, we revised the Statistical Analysis section and added a clear note to Table 5 stating that these categorical predictors were analyzed as coded variables and explaining how the coefficients should be interpreted. We also corrected the Durbin–Watson value (1.896) and clarified that the multivariable regression was conducted using complete cases (n = 332, based on the ANOVA degrees of freedom).

Change made (Discussion- Limitations): We added a limitation statement noting that the model explains only a small proportion of variance in knowledge score and that unmeasured factors (e.g., continuing education exposure and access to evidence-based resources) likely play a larger role.

11. There are still numerous typographical errors and awkward expressions throughout the manuscript. A thorough language revision by a professional English editor is strongly recommended.

Response: Thank you for this comment. We agree that the manuscript required language polishing to improve clarity and readability.

Change made: We performed a comprehensive language revision across the entire manuscript to correct typographical and grammatical errors, improve sentence structure, and resolve awkward phrasing. We also standardized terminology and formatting (e.g., consistent use of KAP terminology, punctuation, and decimal reporting) to enhance overall readability.

Reviewer #4: This is a good study regarding KAP, and helps to inform improvement of pharmacists practice regarding probiotics and prebiotics. Here are my comments:

1. Knowledge score of 50% fall in poor and fair & 70% in fair & knowledgeable as mention in abstract and statistical analysis - 1. Poor Knowledge: 50% or less (≤ 12 points); 2. Fair Knowledge: Scores between 50% and 70% (13-24 points); 3. Knowledgeable: Scores of 70% or higher (≥ 25 points). It should have clear cut off point between these scores.

Response: Thank you for this comment. We would like to clarify that the knowledge cutoffs used in our study are already mutually exclusive and do not overlap at 50% or 70%. However, we agree that the wording in the manuscript could be interpreted ambiguously.

1. Poor Knowledge: ≤ 50% (≤ 17 points)

2. Moderate Knowledge: Scores between >50% and <70% (18-23 points)

3. High Knowledge: Scores of ≥70% ( 24-34 points)

2. As this is cross sectional study please consider include reporting findings accordance to STROBE in study design.

Response: Thank you for this recommendation. We agree that STROBE is the appropriate reporting guideline for cross-sectional studies and improves completeness and transparency.

Change made: We reviewed the manuscript against the STROBE checklist for cross-sectional studies and revised the text to ensure key items are explicitly reported, including: study design and setting, eligibility criteria and recruitment, variables and measurement, efforts to address potential bias, sample size rationale, handling of missing data, and detailed statistical methods.

3. “Questionnaire was reviewed by a panel of specialists in the fields of pharmacy”- Please indicate how many panels were involved for this validity.

Response: Clarification accepted. The panel consisted of four experts.

Change made: We explicitly stated the panel size and composition (4 members)

4. Was the pilot study conducted online to 20 respondents? Please state in procedure

Response: Thank you for this clarification request. The pilot study was conducted by approaching pharmacists in person, and participants completed the draft questionnaire electronically using Google Forms to assess clarity, comprehension, and feasibility.

Change made: We revised the Survey design and validation section to explicitly state the pilot administration method and to clarify that pilot responses (n = 20) were excluded from the final analysis and not included in the final study sample.

“Afterward, a pilot study (n = 20) was conducted by approaching pharmacists in person. Participants completed the draft questionnaire electronically using Google Forms to assess clarity, comprehension, and completion time. Feedback resulted in minor refinements, and pilot responses were excluded from the final dataset and analyses.”

5. Data analysis part- Is there any missing data and how authors handled missing data from respondents?

Response: Thank you for raising this important point. Yes, there were some missing responses for selected variables (e.g., prescription volume and number of employees), as indicated in Table 1. For descriptive analyses, we reported frequencies and percentages and explicitly presented missing categories where applicable. For inferential analyses, we used complete-case (listwise) analysis: participants with missing values in any variable included in the regression model were excluded from that model.

Change made: We have revised the Methods (Statistical analysis) to clearly describe the handling of missing data

6. Results – Please include mean,SD of respondents age for better clarity of demographic respondents.

Response: Thank you for the suggestion. In this survey, age was collected as categorical ranges (20–29, 30–39, 40–49, ≥50) rather than as a continuous variable; therefore, an exact mean and SD cannot be computed from the raw data.

7. Please do not repeat the result values in discussion section. E.g (57.8%), 32.0%, 10.3%

Response: Thank you for this suggestion. We agree that the Discussion should primarily interpret findings rather than restate detailed numerical results that are already presented in the Results section.

Change made: We revised the Discussion to remove repeated reporting of specific percentages (e.g., “57.8%, 32.0%, 10.3%”) and instead focused on interpretation of the overall pattern of findings (e.g., predominance of moderate knowledge, generally positive attitudes, and variability in practices). Where numerical values were necessary for emphasis, we retained only a minimal number of key figures and referred readers to the relevant tables for details.

Attachment Submitted filename: Reviewer report- V2.docx

# Peer review: Decision Letter 2

**DOI:** 10.1371/journal.pone.0350648.r005

**Authors:** Awatif Al-Judaibi, Awatif Al-Judaibi, Awatif Al-Judaibi

5 Apr 2026

PONE-D-25-56829R2

Pharmacists Knowledge, Attitudes, and Practices Regarding Probiotics and Prebiotics: A Cross-Sectional Study from Palestine

PLOS One

Dear Dr. Daraghmeh,

Thank you for submitting your manuscript to PLOS ONE. After careful consideration, we feel that it has merit but does not fully meet PLOS ONE’s publication criteria as it currently stands. Therefore, we invite you to submit a revised version of the manuscript that addresses the points raised during the review process.

Please submit your revised manuscript by May 20 2026 11:59PM. If you will need more time than this to complete your revisions, please reply to this message or contact the journal office at plosone@plos.org. When you're ready to submit your revision, log on to https://www.editorialmanager.com/pone/ and select the 'Submissions Needing Revision' folder to locate your manuscript file.

Please include the following items when submitting your revised manuscript:

- A letter that responds to each point raised by the academic editor and reviewer(s). You should upload this letter as a separate file labeled 'Response to Reviewers'.
- A marked-up copy of your manuscript that highlights changes made to the original version. You should upload this as a separate file labeled 'Revised Manuscript with Track Changes'.
- An unmarked version of your revised paper without tracked changes. You should upload this as a separate file labeled 'Manuscript'.

If you would like to make changes to your financial disclosure, please include your updated statement in your cover letter. Guidelines for resubmitting your figure files are available below the reviewer comments at the end of this letter.

If applicable, we recommend that you deposit your laboratory protocols in protocols.io to enhance the reproducibility of your results. Protocols.io assigns your protocol its own identifier (DOI) so that it can be cited independently in the future. For instructions see: https://journals.plos.org/plosone/s/submission-guidelines#loc-laboratory-protocols. Additionally, PLOS ONE offers an option for publishing peer-reviewed Lab Protocol articles, which describe protocols hosted on protocols.io. Read more information on sharing protocols at https://plos.org/protocols?utm_medium=editorial-email&utm_source=authorletters&utm_campaign=protocols.

As the corresponding author, your ORCID iD is verified in the submission system and will appear in the published article. PLOS supports the use of ORCID, and we encourage all coauthors to register for an ORCID iD and use it as well. Please encourage your coauthors to verify their ORCID iD within the submission system before final acceptance, as unverified ORCID iDs will not appear in the published article. *Only*  the individual author can complete the verification step; PLOS staff *cannot*  verify ORCID iDs on behalf of authors.

We look forward to receiving your revised manuscript.

Kind regards,

Awatif Abid Al-Judaibi, PhD

Academic Editor

PLOS One

Journal Requirements:

If the reviewer comments include a recommendation to cite specific previously published works, please review and evaluate these publications to determine whether they are relevant and should be cited. There is no requirement to cite these works unless the editor has indicated otherwise.

Please review your reference list to ensure that it is complete and correct. If you have cited papers that have been retracted, please include the rationale for doing so in the manuscript text, or remove these references and replace them with relevant current references. Any changes to the reference list should be mentioned in the rebuttal letter that accompanies your revised manuscript. If you need to cite a retracted article, indicate the article’s retracted status in the References list and also include a citation and full reference for the retraction notice.

[Note: HTML markup is below. Please do not edit.]

Reviewers' comments:

Reviewer's Responses to Questions

**Comments to the Author**

1. If the authors have adequately addressed your comments raised in a previous round of review and you feel that this manuscript is now acceptable for publication, you may indicate that here to bypass the “Comments to the Author” section, enter your conflict of interest statement in the “Confidential to Editor” section, and submit your "Accept" recommendation.

Reviewer #1: (No Response)

Reviewer #4: (No Response)

**********

2. Is the manuscript technically sound, and do the data support the conclusions?

The manuscript must describe a technically sound piece of scientific research with data that supports the conclusions. Experiments must have been conducted rigorously, with appropriate controls, replication, and sample sizes. The conclusions must be drawn appropriately based on the data presented.

Reviewer #1: Partly

Reviewer #4: Yes

**********

3. Has the statistical analysis been performed appropriately and rigorously?

Reviewer #1: No

Reviewer #4: Yes

**********

4. Have the authors made all data underlying the findings in their manuscript fully available?

The PLOS Data policy requires authors to make all data underlying the findings described in their manuscript fully available without restriction, with rare exception (please refer to the Data Availability Statement in the manuscript PDF file). The data should be provided as part of the manuscript or its supporting information, or deposited to a public repository. For example, in addition to summary statistics, the data points behind means, medians and variance measures should be available. If there are restrictions on publicly sharing data—e.g. participant privacy or use of data from a third party—those must be specified.

Reviewer #1: Yes

Reviewer #4: Yes

**********

5. Is the manuscript presented in an intelligible fashion and written in standard English?

PLOS ONE does not copyedit accepted manuscripts, so the language in submitted articles must be clear, correct, and unambiguous. Any typographical or grammatical errors should be corrected at revision, so please note any specific errors here.

Reviewer #1: No

Reviewer #4: Yes

**********

6. Review Comments to the Author

Please use the space provided to explain your answers to the questions above. You may also include additional comments for the author, including concerns about dual publication, research ethics, or publication ethics. (Please upload your review as an attachment if it exceeds 20,000 characters)

Reviewer #1: Thanks to the authors for answering my questions and comments in detail. I also have some minor comments and hope that they are helpful to the authors.

1. The reported multivariable linear regression model shows a very poor fit (R² = 0.034; Adjusted R² = −0.002; F(12,319) = 0.936, p = 0.511), indicating that the included predictors explain very little of the variance in knowledge scores. Furthermore, several categorical variables (e.g., education level, province, profile, working setting, and geographic location) were entered as numerically coded single-term predictors rather than as indicator (dummy) variables. As a result, the regression coefficients reflect the expected change in knowledge score per one-unit increase in the coding scale and cannot be interpreted as comparisons between specific categories and a reference group, which may be misleading, especially for nominal variables without inherent ordering. The analysis was conducted using complete-case (listwise deletion, n = 332), which may reduce statistical power and introduce bias if missing data are not completely at random.

The authors are strongly encouraged to recode categorical variables into dummy indicators, clearly specify reference groups, and reconsider the modeling approach, as the current presentation does not allow meaningful interpretation of the effects of categorical predictors.

2. The authors need to ensure consistency across all tables regarding the number of decimal places reported for percentages. A uniform format (e.g., one or two decimal places) should be applied throughout the manuscript to improve clarity and readability.

3. There are still some grammar mistakes throughout the manuscript. A thorough language revision by a professional English editor is strongly recommended. For example:

“Pharmacists were excluded if they are retired pharmacists...”

“The fourth section assess attitudes and practices towards...”

Reviewer #4: Thank you for acknowledge my comments.

Please state explicitly that your study followed guideline of STROBE in your study design paragraph.

**********

7. PLOS authors have the option to publish the peer review history of their article (what does this mean?). If published, this will include your full peer review and any attached files.

If you choose “no”, your identity will remain anonymous but your review may still be made public.

**Do you want your identity to be public for this peer review?**  For information about this choice, including consent withdrawal, please see our Privacy Policy.

Reviewer #1: No

Reviewer #4: No

**********

[NOTE: If reviewer comments were submitted as an attachment file, they will be attached to this email and accessible via the submission site. Please log into your account, locate the manuscript record, and check for the action link "View Attachments". If this link does not appear, there are no attachment files.]

To ensure your figures meet our technical requirements, please review our figure guidelines: https://journals.plos.org/plosone/s/figures

You may also use PLOS’s free figure tool, NAAS, to help you prepare publication quality figures: https://journals.plos.org/plosone/s/figures#loc-tools-for-figure-preparation.

NAAS will assess whether your figures meet our technical requirements by comparing each figure against our figure specifications.

# Author comment: Author response to Decision Letter 3

**DOI:** 10.1371/journal.pone.0350648.r006

9 Apr 2026

Date: 7 Apr. 26

Re: Response to reviewers’ comments on the manuscript:

“Pharmacists Knowledge, Attitudes, and Practices Regarding Probiotics and Prebiotics: A Cross-Sectional Study from Palestine”.

The authors would like to thank the Editor for considering the manuscript for publication in Plos One. The authors would also like to thank the Reviewers for their time and constructive comments and suggestions to improve the quality of the manuscript. The following responses have been prepared to address the comments in a point-by-point fashion.

Reviewer #1:

Thanks to the authors for answering my questions and comments in detail. I also have some minor comments and hope that they are helpful to the authors.

1. The reported multivariable linear regression model shows a very poor fit (R² = 0.034; Adjusted R² = −0.002; F(12,319) = 0.936, p = 0.511), indicating that the included predictors explain very little of the variance in knowledge scores. Furthermore, several categorical variables (e.g., education level, province, profile, working setting, and geographic location) were entered as numerically coded single-term predictors rather than as indicator (dummy) variables. As a result, the regression coefficients reflect the expected change in knowledge score per one-unit increase in the coding scale and cannot be interpreted as comparisons between specific categories and a reference group, which may be misleading, especially for nominal variables without inherent ordering. The analysis was conducted using complete-case (listwise deletion, n = 332), which may reduce statistical power and introduce bias if missing data are not completely at random.

The authors are strongly encouraged to recode categorical variables into dummy indicators, clearly specify reference groups, and reconsider the modeling approach, as the current presentation does not allow meaningful interpretation of the effects of categorical predictors.

Response: We thank the reviewer for this comment. We agree that the initial model, which treated categorical variables as numerically coded predictors, limited interpretability.

Accordingly, we re-conducted the multivariable regression analysis. All categorical variables were entered as factors and dummy-coded with clearly defined reference categories, allowing coefficients to be interpreted as comparisons between categories.

The revised model demonstrated modest explanatory power and was statistically significant overall (R² = 0.10; adjusted R² = −0.04; F(22, 324) = 1.63; p = 0.04). Several variables were significantly associated with knowledge score, including age, years of experience, working hours, and prescription volume.

Changes: We have updated the Abstract, Methods, Results, Table 5, and Discussion accordingly, and clarified the use of complete-case analysis as a limitation.

2. The authors need to ensure consistency across all tables regarding the number of decimal places reported for percentages. A uniform format (e.g., one or two decimal places) should be applied throughout the manuscript to improve clarity and readability.

Response: We thank the reviewer for this suggestion. We have revised the manuscript to improve consistency in the presentation of percentages across tables and text.

3. There are still some grammar mistakes throughout the manuscript. A thorough language revision by a professional English editor is strongly recommended. For example:

“Pharmacists were excluded if they are retired pharmacists...”

“The fourth section assess attitudes and practices towards...”

Response: We thank the reviewer for this comment. The manuscript has been carefully revised to improve grammar, clarity, and readability. Sentence structure, verb tense, and phrasing inconsistencies have been corrected throughout the manuscript.

Reviewer #4:

Thank you for acknowledge my comments.

Please state explicitly that your study followed guideline of STROBE in your study design paragraph.

Response: We thank the reviewer for this comment. We have now explicitly stated that the study was designed and reported in accordance with the STROBE guidelines for cross-sectional studies.

Attachment Submitted filename: Reviewer report responses.docx

# Peer review: Decision Letter 3

**DOI:** 10.1371/journal.pone.0350648.r007

**Authors:** Awatif Al-Judaibi, Awatif Al-Judaibi, Awatif Al-Judaibi, Awatif Al-Judaibi

16 Apr 2026

PONE-D-25-56829R3

Pharmacists Knowledge, Attitudes, and Practices Regarding Probiotics and Prebiotics: A Cross-Sectional Study from Palestine

PLOS One

Dear Dr. Dala N Daraghmeh,

Thank you for submitting your manuscript to PLOS ONE. After careful consideration, we feel that it has merit but does not fully meet PLOS ONE’s publication criteria as it currently stands. Therefore, we invite you to submit a revised version of the manuscript that addresses the points raised during the review process.

Please submit your revised manuscript by May 31 2026 11:59PM. If you will need more time than this to complete your revisions, please reply to this message or contact the journal office at plosone@plos.org. When you're ready to submit your revision, log on to https://www.editorialmanager.com/pone/ and select the 'Submissions Needing Revision' folder to locate your manuscript file.

Please include the following items when submitting your revised manuscript:

- A letter that responds to each point raised by the academic editor and reviewer(s). You should upload this letter as a separate file labeled 'Response to Reviewers'.
- A marked-up copy of your manuscript that highlights changes made to the original version. You should upload this as a separate file labeled 'Revised Manuscript with Track Changes'.
- An unmarked version of your revised paper without tracked changes. You should upload this as a separate file labeled 'Manuscript'.

If you would like to make changes to your financial disclosure, please include your updated statement in your cover letter. Guidelines for resubmitting your figure files are available below the reviewer comments at the end of this letter.

If applicable, we recommend that you deposit your laboratory protocols in protocols.io to enhance the reproducibility of your results. Protocols.io assigns your protocol its own identifier (DOI) so that it can be cited independently in the future. For instructions see: https://journals.plos.org/plosone/s/submission-guidelines#loc-laboratory-protocols. Additionally, PLOS ONE offers an option for publishing peer-reviewed Lab Protocol articles, which describe protocols hosted on protocols.io. Read more information on sharing protocols at https://plos.org/protocols?utm_medium=editorial-email&utm_source=authorletters&utm_campaign=protocols.

As the corresponding author, your ORCID iD is verified in the submission system and will appear in the published article. PLOS supports the use of ORCID, and we encourage all coauthors to register for an ORCID iD and use it as well. Please encourage your coauthors to verify their ORCID iD within the submission system before final acceptance, as unverified ORCID iDs will not appear in the published article. *Only*  the individual author can complete the verification step; PLOS staff *cannot*  verify ORCID iDs on behalf of authors.

We look forward to receiving your revised manuscript.

Kind regards,

Awatif Abid Al-Judaibi, PhD

Academic Editor

PLOS One

Journal Requirements:

If the reviewer comments include a recommendation to cite specific previously published works, please review and evaluate these publications to determine whether they are relevant and should be cited. There is no requirement to cite these works unless the editor has indicated otherwise.

Please review your reference list to ensure that it is complete and correct. If you have cited papers that have been retracted, please include the rationale for doing so in the manuscript text, or remove these references and replace them with relevant current references. Any changes to the reference list should be mentioned in the rebuttal letter that accompanies your revised manuscript. If you need to cite a retracted article, indicate the article’s retracted status in the References list and also include a citation and full reference for the retraction notice.

[Note: HTML markup is below. Please do not edit.]

Reviewers' comments:

Reviewer's Responses to Questions

**Comments to the Author**

1. If the authors have adequately addressed your comments raised in a previous round of review and you feel that this manuscript is now acceptable for publication, you may indicate that here to bypass the “Comments to the Author” section, enter your conflict of interest statement in the “Confidential to Editor” section, and submit your "Accept" recommendation.

Reviewer #1: (No Response)

**********

2. Is the manuscript technically sound, and do the data support the conclusions?

The manuscript must describe a technically sound piece of scientific research with data that supports the conclusions. Experiments must have been conducted rigorously, with appropriate controls, replication, and sample sizes. The conclusions must be drawn appropriately based on the data presented.

Reviewer #1: Yes

**********

3. Has the statistical analysis been performed appropriately and rigorously?

Reviewer #1: No

**********

4. Have the authors made all data underlying the findings in their manuscript fully available?

The PLOS Data policy requires authors to make all data underlying the findings described in their manuscript fully available without restriction, with rare exception (please refer to the Data Availability Statement in the manuscript PDF file). The data should be provided as part of the manuscript or its supporting information, or deposited to a public repository. For example, in addition to summary statistics, the data points behind means, medians and variance measures should be available. If there are restrictions on publicly sharing data—e.g. participant privacy or use of data from a third party—those must be specified.

Reviewer #1: Yes

**********

5. Is the manuscript presented in an intelligible fashion and written in standard English?

PLOS ONE does not copyedit accepted manuscripts, so the language in submitted articles must be clear, correct, and unambiguous. Any typographical or grammatical errors should be corrected at revision, so please note any specific errors here.

Reviewer #1: Yes

**********

6. Review Comments to the Author

Please use the space provided to explain your answers to the questions above. You may also include additional comments for the author, including concerns about dual publication, research ethics, or publication ethics. (Please upload your review as an attachment if it exceeds 20,000 characters)

Reviewer #1: The authors conducted a multivariable linear regression with a large number of covariates; however, several concerns regarding model performance and interpretability remain.

First, the model's reported explanatory power is very limited (R² = 0.10), and, more importantly, the adjusted R² is negative (−0.04). This indicates that the fitted model does not improve upon a null model with no predictors and suggests potential overfitting due to the inclusion of a large number of covariates relative to the sample size. Including 22 predictors may have introduced substantial noise, thereby reducing the model’s overall reliability.

Second, although the overall F-test was statistically significant (p = 0.04), this result should be interpreted with caution. A statistically significant F-test in the presence of a negative adjusted R² may reflect marginal effects driven by a small subset of variables rather than a well-performing model. Therefore, the model's practical significance and robustness are questionable.

Third, the authors report several statistically significant predictors; however, given the low explanatory power and potential overfitting, these associations may not be stable. It would be helpful to assess the robustness of these findings using alternative approaches, such as variable selection techniques or model reduction strategies.

To improve the analysis, the authors are encouraged to:

1. Consider reducing the number of covariates based on theoretical justification or data-driven approaches.

2. Evaluate model stability (e.g., through sensitivity analyses or penalized regression methods).

3. Report additional diagnostics (e.g., multicollinearity, residual analysis) to support model validity.

Overall, while the topic is relevant, the current multivariable model requires further refinement to ensure valid and interpretable conclusions.

**********

7. PLOS authors have the option to publish the peer review history of their article (what does this mean?). If published, this will include your full peer review and any attached files.

If you choose “no”, your identity will remain anonymous but your review may still be made public.

**Do you want your identity to be public for this peer review?**  For information about this choice, including consent withdrawal, please see our Privacy Policy.

Reviewer #1: No

**********

[NOTE: If reviewer comments were submitted as an attachment file, they will be attached to this email and accessible via the submission site. Please log into your account, locate the manuscript record, and check for the action link "View Attachments". If this link does not appear, there are no attachment files.]

To ensure your figures meet our technical requirements, please review our figure guidelines: https://journals.plos.org/plosone/s/figures

You may also use PLOS’s free figure tool, NAAS, to help you prepare publication quality figures: https://journals.plos.org/plosone/s/figures#loc-tools-for-figure-preparation.

NAAS will assess whether your figures meet our technical requirements by comparing each figure against our figure specifications.

# Author comment: Author response to Decision Letter 4

**DOI:** 10.1371/journal.pone.0350648.r008

20 Apr 2026

Date: 18 Apr. 26

Re: Response to reviewers’ comments on the manuscript:

“Pharmacists Knowledge, Attitudes, and Practices Regarding Probiotics and Prebiotics: A Cross-Sectional Study from Palestine”.

The authors would like to thank the Editor for considering the manuscript for publication in Plos One. The authors would also like to thank the Reviewers for their time and constructive comments and suggestions to improve the quality of the manuscript. The following responses have been prepared to address the comments in a point-by-point fashion.

Reviewer Comment:

Reviewer #1: The authors conducted a multivariable linear regression with a large number of covariates; however, several concerns regarding model performance and interpretability remain.

First, the model's reported explanatory power is very limited (R² = 0.10), and, more importantly, the adjusted R² is negative (−0.04). This indicates that the fitted model does not improve upon a null model with no predictors and suggests potential overfitting due to the inclusion of a large number of covariates relative to the sample size. Including 22 predictors may have introduced substantial noise, thereby reducing the model’s overall reliability.

Second, although the overall F-test was statistically significant (p = 0.04), this result should be interpreted with caution. A statistically significant F-test in the presence of a negative adjusted R² may reflect marginal effects driven by a small subset of variables rather than a well-performing model. Therefore, the model's practical significance and robustness are questionable.

Third, the authors report several statistically significant predictors; however, given the low explanatory power and potential overfitting, these associations may not be stable. It would be helpful to assess the robustness of these findings using alternative approaches, such as variable selection techniques or model reduction strategies.

To improve the analysis, the authors are encouraged to:

1. Consider reducing the number of covariates based on theoretical justification or data-driven approaches.

2. Evaluate model stability (e.g., through sensitivity analyses or penalized regression methods).

3. Report additional diagnostics (e.g., multicollinearity, residual analysis) to support model validity.

Response:

We thank the reviewer for this detailed and insightful critique. We fully acknowledge the concerns raised regarding model overfitting, interpretability, and validity. In response, we have substantially revised the multivariable analysis to address these issues in a rigorous and systematic manner.

First, to address overfitting and improve interpretability, we replaced the original full model (22 covariates) with a reduced model including only variables selected based on strong theoretical relevance and empirical support from prior literature. The revised model includes probiotic/prebiotic education, age, gender, prescription volume, and practice setting. This approach aligns with recommended practices for parsimonious model specification and avoids unnecessary inclusion of weak or redundant predictors.

Second, the revised model demonstrated improved performance, with a positive adjusted R² (adjusted R² = 0.04) compared to the previously negative value (−0.04), indicating that the model now provides explanatory value beyond a null model. Although the overall explanatory power remains modest (R² = 0.06), this is consistent with the exploratory nature of KAP studies, where knowledge outcomes are influenced by multiple unmeasured behavioral and contextual factors.

Third, we conducted additional diagnostic assessments to ensure model validity. Multicollinearity was evaluated using variance inflation factors (VIF), with all values approximately equal to 1, indicating no evidence of collinearity among predictors. Residual diagnostics were also examined and did not suggest violations of model assumptions.

Fourth, to assess robustness, we performed sensitivity analyses by applying robust standard errors (HC1) to account for potential heteroscedasticity. The results remained consistent with the primary model, particularly with respect to the association between probiotic education and knowledge score, supporting the stability of the results.

Finally, we have revised the interpretation throughout the manuscript to reflect the limited explanatory power of the model and to avoid overstatement of findings. The results are now explicitly presented as exploratory associations rather than definitive predictors, consistent with the cross-sectional design and the descriptive purpose of the study.

Attachment Submitted filename: Response report 18042026.docx

# Peer review: Decision Letter 4

**DOI:** 10.1371/journal.pone.0350648.r009

**Authors:** Awatif Al-Judaibi, Awatif Al-Judaibi, Awatif Al-Judaibi, Awatif Al-Judaibi, Awatif Al-Judaibi

17 May 2026

Pharmacists Knowledge, Attitudes, and Practices Regarding Probiotics and Prebiotics: A Cross-Sectional Study from Palestine

PONE-D-25-56829R4

Dear Dr. Dala N Daraghmeh,

We’re pleased to inform you that your manuscript has been judged scientifically suitable for publication and will be formally accepted for publication once it meets all outstanding technical requirements.

Within one week, you’ll receive an e-mail detailing the required amendments. When these have been addressed, you’ll receive a formal acceptance letter and your manuscript will be scheduled for publication.

An invoice will be generated when your article is formally accepted. Please note, if your institution has a publishing partnership with PLOS and your article meets the relevant criteria, all or part of your publication costs will be covered. Please make sure your user information is up-to-date by logging into Editorial Manager at Editorial Manager® and clicking the ‘Update My Information' link at the top of the page. For questions related to billing, please contact billing support.

If your institution or institutions have a press office, please notify them about your upcoming paper to help maximize its impact. If they’ll be preparing press materials, please inform our press team as soon as possible -- no later than 48 hours after receiving the formal acceptance. Your manuscript will remain under strict press embargo until 2 pm Eastern Time on the date of publication. For more information, please contact onepress@plos.org.

Kind regards,

Awatif Abid Al-Judaibi, PhD

Academic Editor

PLOS One

Reviewers' comments:

Reviewer's Responses to Questions

**Comments to the Author**

1. If the authors have adequately addressed your comments raised in a previous round of review and you feel that this manuscript is now acceptable for publication, you may indicate that here to bypass the “Comments to the Author” section, enter your conflict of interest statement in the “Confidential to Editor” section, and submit your "Accept" recommendation.

Reviewer #1: All comments have been addressed

**********

2. Is the manuscript technically sound, and do the data support the conclusions?

The manuscript must describe a technically sound piece of scientific research with data that supports the conclusions. Experiments must have been conducted rigorously, with appropriate controls, replication, and sample sizes. The conclusions must be drawn appropriately based on the data presented.

Reviewer #1: Yes

**********

3. Has the statistical analysis been performed appropriately and rigorously?

Reviewer #1: Yes

**********

4. Have the authors made all data underlying the findings in their manuscript fully available?

The PLOS Data policy requires authors to make all data underlying the findings described in their manuscript fully available without restriction, with rare exception (please refer to the Data Availability Statement in the manuscript PDF file). The data should be provided as part of the manuscript or its supporting information, or deposited to a public repository. For example, in addition to summary statistics, the data points behind means, medians and variance measures should be available. If there are restrictions on publicly sharing data—e.g. participant privacy or use of data from a third party—those must be specified.

Reviewer #1: Yes

**********

5. Is the manuscript presented in an intelligible fashion and written in standard English?

PLOS ONE does not copyedit accepted manuscripts, so the language in submitted articles must be clear, correct, and unambiguous. Any typographical or grammatical errors should be corrected at revision, so please note any specific errors here.

Reviewer #1: Yes

**********

6. Review Comments to the Author

Please use the space provided to explain your answers to the questions above. You may also include additional comments for the author, including concerns about dual publication, research ethics, or publication ethics. (Please upload your review as an attachment if it exceeds 20,000 characters)

Reviewer #1: Thank the authors for their detailed response to my comments and for revising their manuscript. From my perspective, this revised manuscript is of high quality for publication. Best wishes to the authors.

**********

7. PLOS authors have the option to publish the peer review history of their article (what does this mean?). If published, this will include your full peer review and any attached files.

If you choose “no”, your identity will remain anonymous but your review may still be made public.

**Do you want your identity to be public for this peer review?**  For information about this choice, including consent withdrawal, please see our Privacy Policy.

Reviewer #1: No

**********

# Editor report: Acceptance letter

**DOI:** 10.1371/journal.pone.0350648.r010

**Authors:** Awatif Al-Judaibi, Awatif Al-Judaibi, Awatif Al-Judaibi, Awatif Al-Judaibi, Awatif Al-Judaibi

PONE-D-25-56829R4

PLOS One

Dear Dr. Daraghmeh,

I'm pleased to inform you that your manuscript has been deemed suitable for publication in PLOS One. Congratulations! Your manuscript is now being handed over to our production team.

At this stage, our production department will prepare your paper for publication. This includes ensuring the following:

* All references, tables, and figures are properly cited

* All relevant supporting information is included in the manuscript submission,

* There are no issues that prevent the paper from being properly typeset

You will receive further instructions from the production team, including instructions on how to review your proof when it is ready. Please keep in mind that we are working through a large volume of accepted articles, so please give us a few days to review your paper and let you know the next and final steps.

Lastly, if your institution or institutions have a press office, please let them know about your upcoming paper now to help maximize its impact. If they'll be preparing press materials, please inform our press team within the next 48 hours. Your manuscript will remain under strict press embargo until 2 pm Eastern Time on the date of publication. For more information, please contact onepress@plos.org.

You will receive an invoice from PLOS for your publication fee after your manuscript has reached the completed accept phase. If you receive an email requesting payment before acceptance or for any other service, this may be a phishing scheme. Learn how to identify phishing emails and protect your accounts at https://explore.plos.org/phishing.

If we can help with anything else, please email us at customercare@plos.org.

Thank you for submitting your work to PLOS ONE and supporting open access.

Kind regards,

PLOS ONE Editorial Office Staff

on behalf of

Professor Awatif Abid Al-Judaibi

Academic Editor

PLOS One
